# KRAS mRNA Spleen‐Targeting Lipid Nanoparticles Synergize with Irinotecan Silicasomes to Robustly Augment the Cancer Immunity Cycle in Pancreatic Cancer

**DOI:** 10.1002/advs.202504886

**Published:** 2025-06-11

**Authors:** Lijia Luo, Xiang Wang, Yu‐Pei Liao, Andre E. Nel

**Affiliations:** ^1^ Division of Nanomedicine Department of Medicine University of California Los Angeles CA 90095 USA; ^2^ California NanoSystems Institute University of California Los Angeles CA 90095 USA

**Keywords:** cancer immunity cycle, dual drug silicasome, immunotherapy, lipid nanoparticles, mRNA delivery, pancreatic cancer, TLR agonists

## Abstract

Pancreatic ductal adenocarcinoma (PDAC) remains one of the most lethal malignancies due to its immunosuppressive tumor microenvironment. It is hypothesized that overcoming these barriers requires a dual approach: inducing immunogenic tumor cell death (ICD) and enhancing the cancer immunity cycle by exogenous neoantigen targeting on the spleen. In this study, a novel strategy is presented combining irinotecan‐loaded silicasomes with spleen‐targeting lipid nanoparticles (LNPs) carrying KRAS^G12D^ mRNA and the toll‐like receptor 7/8 (TLR7/8) agonist 3M‐052. The goal is to establish a cancer immunity cycle by promoting endogenous tumor antigen release by the generation of KRAS‐specific cytotoxic T cells. Using an orthotopic PDAC mouse model, it is demonstrated that this dual‐platform approach significantly reduces tumor burden and extends survival compared to monotherapies. Bulk RNA sequencing and gene expression analyses further reveal synergy between the immune responses at the primary tumor site and the spleen, including maximal upregulation of apoptosis‐related genes, endoplasmic reticulum stress pathways, antigen presentation pathways, and T cell activation markers. These findings indicate that the combinatorial strategy effectively bridges innate and adaptive immunity. In conclusion, this study highlights the potential of nanocarrier‐based immunotherapy to enhance PDAC immunity by integrating ICD induction with systemic immune reprogramming, offering a promising avenue for improving treatment outcomes.

## Graphical abstract

To overcome pancreatic cancer's immunosuppressive barriers, this study explores a dual approach: triggering immunogenic tumor cell death at the primary tumor site with irinotecan‐loaded silicasomes and enhancing immune activation in the spleen using LNPs encapsulating tumor antigen mRNA and a TLR7/8 agonist. This nanocarrier‐based strategy enhances antigen presentation, T cell activation, and tumor reduction, offering a promising cancer immunotherapy strategy.

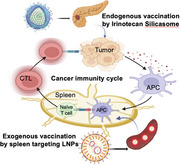

## 1 Introduction

PDAC remains one of the deadliest malignancies, characterized by its late presentation, aggressive biology, dense stromal microenvironment, and significant resistance to conventional therapies.^[^ ^1^ ^]^ Despite advances in surgical techniques and chemotherapeutic regimens, the overall survival rate for PDAC patients remains dismal.^[^ ^2^ ^]^ This therapeutic challenge is, in part, attributable to the immunosuppressive tumor microenvironment that effectively blunts the anti‐PDAC immune response, despite the availability of new treatment modalities such as immune checkpoint blockage.^[^ ^3^ ^]^ Thus, while immunotherapy has emerged as a promising avenue for treating various cancers, its success in PDAC has been limited due to suboptimal activation of the immune system within the tumor immune microenvironment (TIME).^[^ ^3^, ^4^ ^]^ To overcome this barrier, innovative strategies that precisely modulate the immune response are critically needed. Our previous work has focused on developing nanocarrier‐based approaches to reprogram the PDAC immune landscape, thereby enhancing the efficacy of immunotherapy.^[^ ^3^, ^5^, ^6^, ^7^, ^8^, ^9^ ^]^

To date, our research has demonstrated considerable promise using two distinct nanocarrier platforms. The first employs mesoporous silica nanoparticles (MSNPs) cloaked with a lipid bilayer ‐ termed “silicasomes” – designed to deliver irinotecan directly to PDAC tumors and induce immunogenic cell death.^[^ ^6^, ^7^, ^9^ ^]^ The second platform utilizes cationic LNPs engineered to deliver a G12D KRAS mutant mRNA plus a STING agonist, a strategy employed to reprogram the tolerogenic liver immune environment to prevent and treat metastatic PDAC spread.^[^ ^5^ ^]^ These innovative platforms form the cornerstone of our integrated strategy in this communication to disrupt the immunosuppressive PDAC milieu and reinvigorate antitumor immunity.

**Figure 1:**
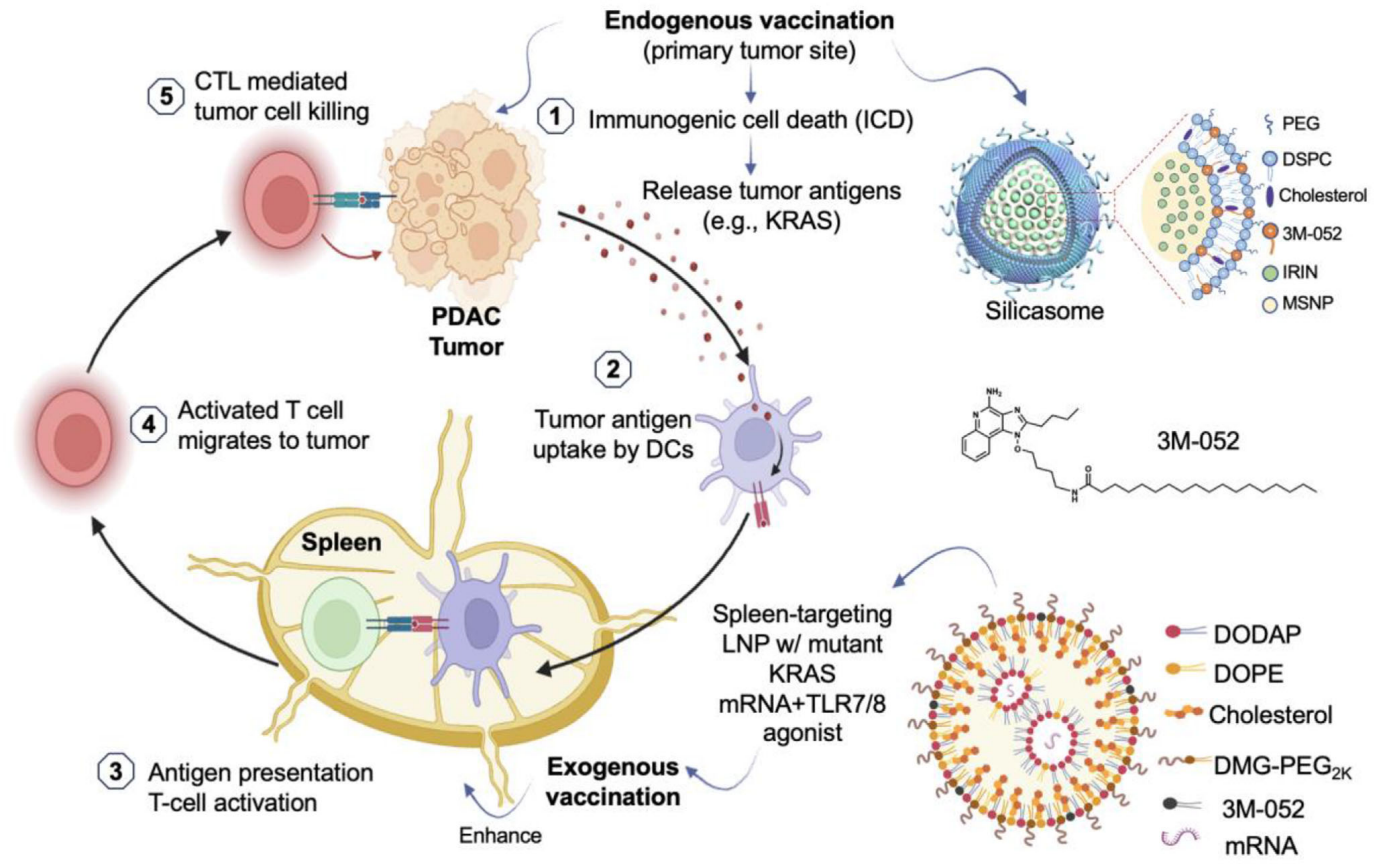
Schematic to explain a dual nanocarrier strategy for enhancing PDAC Tumor Immunity. The schematic illustrates a dual vaccination strategy, hypothesized to enhance the PDAC tumor immunity cycle by integrating endogenous and exogenous vaccination approaches. This strategy employs two distinct nanocarriers to synergistically reinforce CTL activation against PDAC. Endogenous Vaccination: Chemotherapeutic agents such as irinotecan induce ICD, triggering the release of tumor antigens and DAMPs, including HMGB1, ATP, and CRT. These signals promote the uptake of endogenous tumor antigens and activation of APC, which in turn prime CTLs. The proposed use of silicasome‐encapsulated irinotecan ‐ incorporating the TLR7/8 agonist 3M‐052 ‐ further enhances APC activation and antigen presentation, thereby strengthening the immune response. Exogenous Vaccination: In parallel, spleen‐targeting LNPs delivering mutant KRAS mRNA and a TLR7/8 agonist are proposed to augment the immune response by increasing the frequency of CTLs within the spleen. These activated tumor‐specific CTLs subsequently migrate to the primary tumor site, where they mediate effective cancer cell killing. By integrating these two vaccination mechanisms, the strategy aims to optimize the cancer immunity cycle, leading to a more robust and sustained anti‐tumor immune response.

Building on our previous work, we sought to extend our discovery by demonstrating that silicasomes not only enhance the targeted delivery of irinotecan to PDAC tumors but also robustly trigger immunogenic cell death (ICD), functioning as an endogenous vaccination process.^[^ ^5^, ^6^, ^7^, ^8^, ^9^, ^10^, ^11^ ^]^ ICD is a form of cell death that, unlike traditional apoptosis, actively stimulates an immune response by releasing damage‐associated molecular patterns (DAMPs) and by enhancing the uptake of neoantigens by antigen‐presenting cells (APC), which subsequently migrate to the spleen and secondary lymphoid organs.^[^ ^10^, ^11^ ^]^ The silicasome‐mediated delivery of irinotecan facilitates the release of key DAMPs, such as calreticulin (CRT), high‐mobility group box 1 (HMGB1), and ATP, which collectively promote the maturation and activation of APC (Figure S4, Supporting Information).^[^ ^6^ ^]^ This process triggers a cascade of immune events, ultimately leading to the priming of cytotoxic T lymphocytes (CTLs) in the spleen (**Figure** **1**). These activated CTLs are then recruited back to the tumor site by a chemokine mediated process, completing a critical step in the cancer immunity cycle.^[^ ^12^ ^]^ Simultaneously, our work with cationic LNPs has demonstrated that delivery of a mutant KRAS mRNA can reprogram immune responses in immune‐privileged organs like the liver by reversing tolerogenic signals.^[^ ^5^ ^]^ Initially designed to exploit the tolerogenic properties of liver APC, the inclusion of a STING agonist in cationic LNPs reprogrammed this tolerogenic environment, enhancing the immune response to a PDAC neoantigen and converting the immunosuppressive niche into one that supports antitumor immunity.^[^ ^5^ ^]^ The ability to modulate both local (tumor) and systemic (metastatic) immune responses offers a powerful approach to tackling the multifaceted nature of PDAC.

Despite the promise of these individual strategies, each approach has inherent limitations when used alone. While silicasomes effectively induce ICD, their therapeutic impact is hindered by immunosuppressive feedback mechanisms within the TIME that can attenuate both chemo‐ and immunotherapy responses.^[^ ^3^ ^]^ Similarly, while cationic LNPs effectively reprogram the immune environment in the liver, they may not exert a sufficient impact on the primary tumor site to fully impact the PDAC immunity cycle. This limitation arises, in part, from differences in APC mechanisms between the liver and secondary lymphoid organs. Recognizing these challenges, we hypothesized that combining irinotecan‐loaded silicasomes, which locally induce ICD, with spleen‐targeting LNPs delivering KRAS mRNA may be able to synergistically activate the PDAC cancer immunity cycle (Figure 1). Furthermore, we proposed that incorporating a TLR7/8 agonist into both nanocarriers could further amplify the immune response at the cancer site, as previously demonstrated by us.^[^ ^7^ ^]^ The rationale behind this combination is supported by preliminary transcriptomic analyses, suggesting that simultaneous application of these two platforms leads to an enhanced expression of key genes involved in antigen processing, presentation, and T cell activation. By harnessing the complementary mechanisms of endogenous vaccination (via ICD) and exogenous vaccination (via mRNA delivery), our strategy aims to create a robust immunostimulatory environment that not only facilitates direct tumor cell killing but also establishes a durable response against PDAC (Figure 1).

In the current study, we present a comprehensive investigation into the synergistic potential of two distinct nanocarrier systems in an orthotopic KPC model of pancreatic cancer. By combining irinotecan‐loaded silicasomes with spleen‐targeting LNPs carrying KRAS mRNA, our aim is to enhance the activation of the cancer immunity cycle and overcome the formidable immunosuppressive barriers of PDAC. We will demonstrate that this combinatorial approach achieved superior tumor regression compared to monotherapies in addition to prolonging survival. Furthermore, bulk RNA sequencing and gene expression analyses provided molecular insights into the immune mechanisms underpinning this synergy. This includes the increased expression of sets of genes that reflect bridging of innate and adaptive immunity. By integrating localized ICD induction with systemic immune reprogramming, our findings highlight the potential of this nanocarrier‐based approach to reshape the tumor microenvironment and drive a more effective antitumor response in pancreatic cancer.

## 2 Results

### 2.1 Development of a Spleen‐Targeting LNP Platform for Delivering mRNA Constructs and a TLR7/8 Agonist (3M‐052)

**Figure 2:**
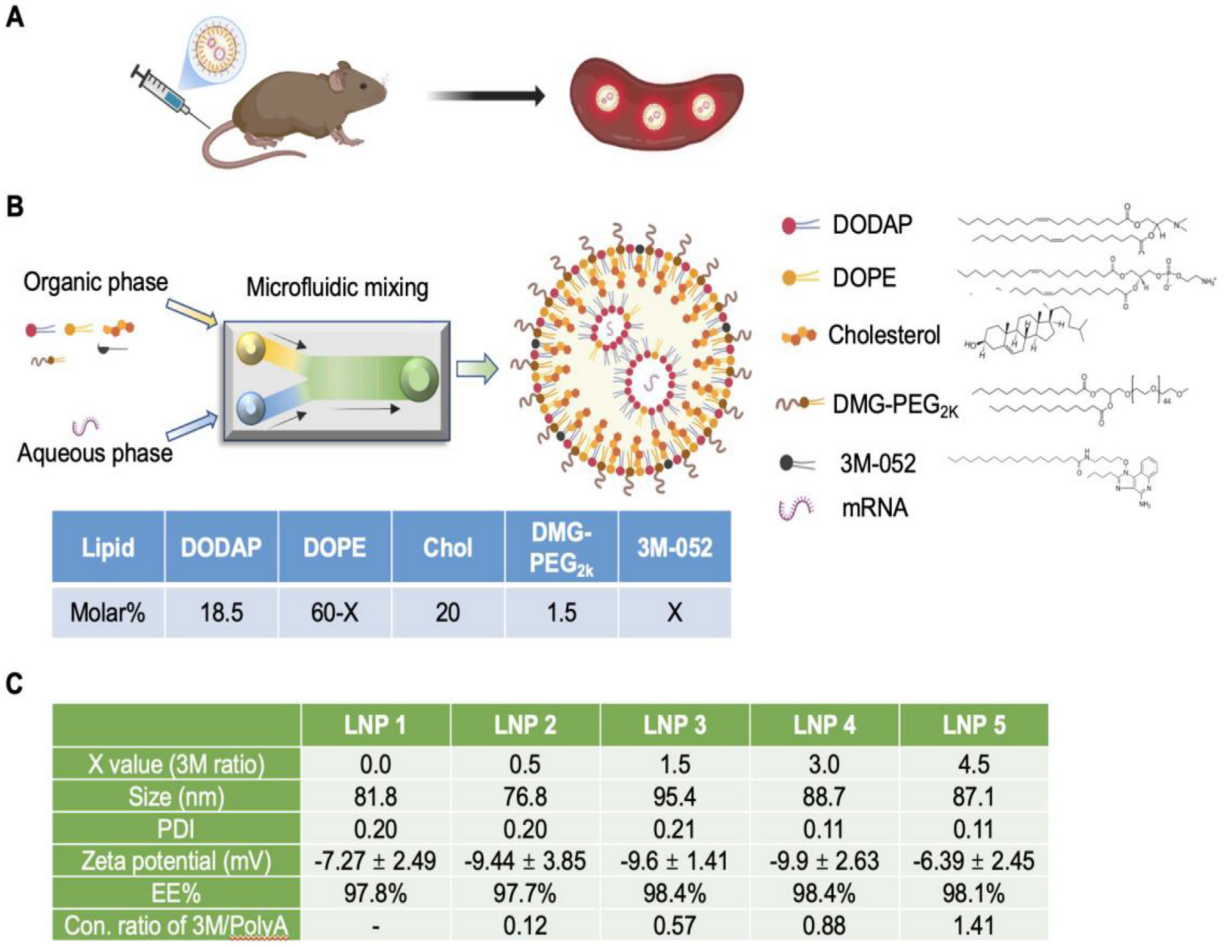
Development and characterization of the spleen‐targeting LNP platform. A) Schematic to illustrate the use of a cationic LNP platform designed to co‐deliver mRNA and a TLR7/8 agonist (3M‐052) to the spleen after IV administration. B) Diagram of LNP synthesis by a microfluidic system. The organic phase, composed of DODAP, DOPE, cholesterol, DMG‐PEG_2k_, and 3M‐052, was mixed with the aqueous phase containing the mRNA. The molar ratios of lipid components are specified in the table. C) Physicochemical characterization of LNPs formulated with varying molar percentages of 3M‐052 (X refers to values of 0, 0.5, 1.5, 3, and 4.5 molar %). The characterization was done for size, PDI, zeta potential, encapsulation efficiency (EE%), and the 3M‐052 to PolyA concentration ratio. The concentration of poly A was determined using the RiboGreen assay, while the concentration of 3M‐052 was measured by UV–vis absorbance at 320 nm. Based on these measurements, the 3M‐052 to Poly A concentration ratio was calculated, with 3M‐LNPs containing 4.5% 3M‐052 (used in animal experiments) having a ratio of 1.4, meaning that when mice were injected with 1.25 mg kg^−1^ mRNA, the corresponding 3M‐052 dose was automatically calculated as 1.75 mg kg^−1^.

Our goal was to develop a nanocarrier capable of delivering mRNA constructs to APC in the spleen while simultaneously co‐delivering a TLR7/8 pathway agonist (**Figure** **2**A). To achieve this, we designed LNPs that encapsulate mRNA within their core, with additional incorporation of a TLR7/8 agonist as an immune adjuvant. The agonist selected for its potency was 3M‐052, capable of activating dendritic cells and augmenting CTL responses, essential requirements to overcome the immunosuppressive TIME in pancreatic cancer.^[^ ^7^, ^13^ ^]^ Its C18 lipid tail (Figure 1) allows stable incorporation into lipid nanoparticles, providing sustained co‐delivery of the adjuvant while avoiding systemic toxicity. 3M‐052 has also been successfully employed in various nanoparticle platforms for cancer and infectious disease immunotherapy, supporting its inclusion in our spleen‐targeted KRAS mRNA lipid nanoparticles designed to drive effective T cell priming and tumor infiltration.^[^ ^7^, ^14^, ^15^, ^16^, ^17^ ^]^ The LNPs were synthesized using a microfluidic system. In this process, an organic phase—consisting of a lipid mixture (DODAP/DOPE/cholesterol/DMG‐PEG2000 at a molar ratio of 18.5:60:20:1.5) with or without 3M‐052 dissolved in ethanol—was combined with an aqueous phase containing mRNA constructs in RNase‐free sodium acetate buffer (Figure 2B).

Previous studies have shown that mRNA‐loaded LNPs formulated with the commercially available pH‐sensitive cationic lipid DODAP ((1,2‐dioleoyl‐3‐dimethylammonium propane) exhibit spleen‐targeting capability after IV administration when combined with the helper lipid DOPE (1,2‐dioleoyl‐sn‐glycero‐3‐phosphoethanolamine).^[^ ^18^ ^]^ This specific lipid composition facilitates the delivery of antigen epitope‐expressing mRNA to the APC‐rich spleen (Figure S2, Supporting Information), thereby enhancing antigen presentation in the cancer immunity cycle (Figure 1). To assess the ability of the spleen‐targeting LNP system to incorporate a TLR7/8 agonist, we synthesized a series of LNPs containing a random RNA construct (PolyA) and varying amounts of 3M‐052 (0% to 4.5% of total lipids). This was achieved by partially substituting DOPE with the lipid‐tailed agonist, as detailed in Figure 2B. After synthesis, the LNP/PolyA formulations, containing varying molar ratios of 3M‐052, were dialyzed and characterized (Figure 2C). Hydrodynamic size measurements revealed that the LNPs ranged from 76.8 to 95.4 nm, with corresponding zeta potentials between ‐6.39 and ‐9.9 mV. Encapsulation efficiency was consistently above 97% across all formulations. Additionally, the concentration ratio of 3M‐052 to PolyA increased proportionally with the molar percentage of 3M‐052. Based on the amount required for subsequent animal studies, LNPs containing 4.5% 3M‐052 were selected for further experimentation, yielding a 3M‐052/PolyA concentration ratio of 1.41.

**Figure 3:**
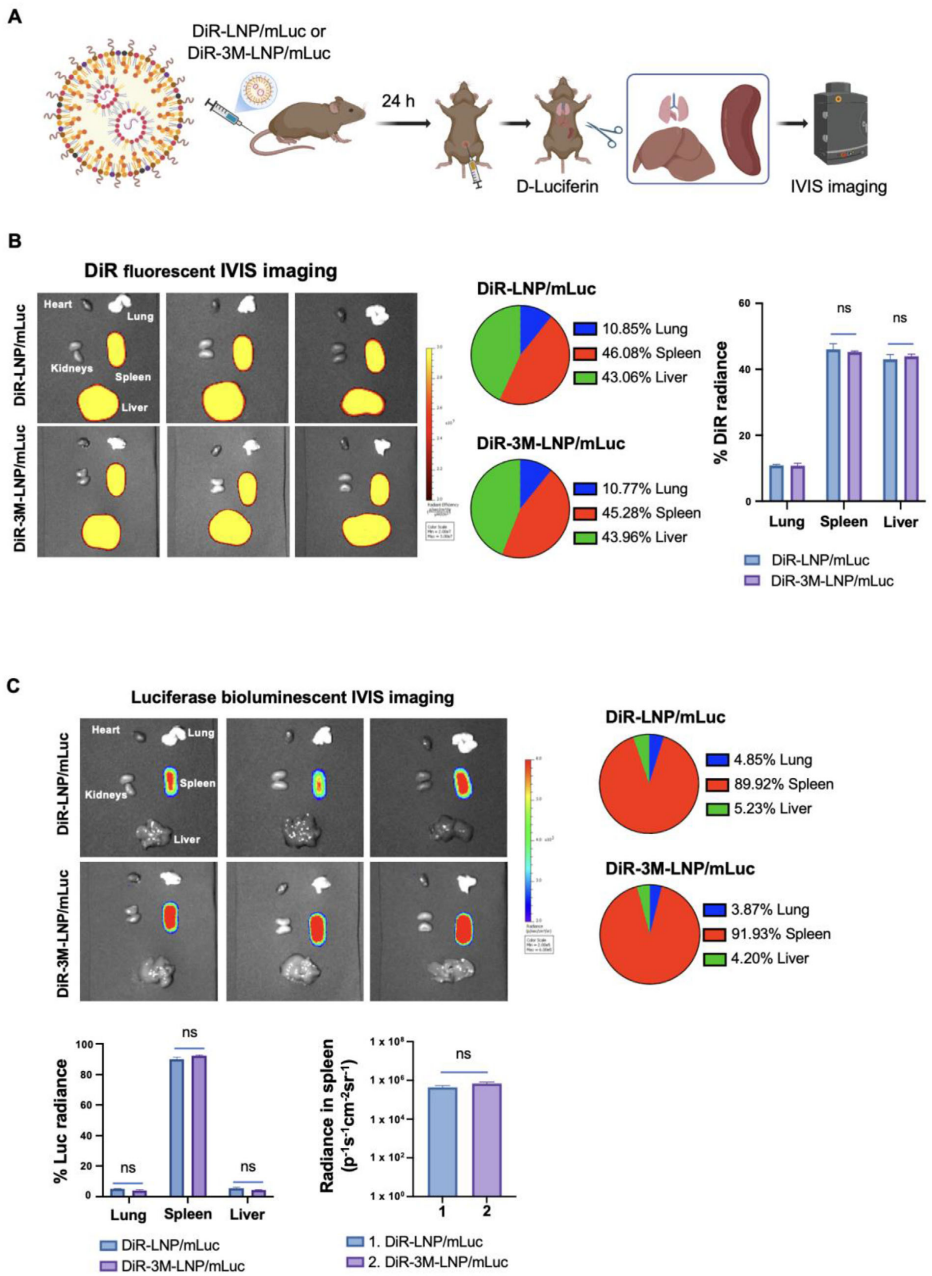
Evaluation of Spleen Targeting and mRNA Expression by the LNP Platform. To assess the spleen‐targeting ability and mRNA expression efficiency of the LNP platform, DiR‐labeled LNPs, with or without 4.5% 3M‐052, were formulated to deliver mLuc mRNA and designated as DiR‐LNP/mLuc and DiR‐3M‐LNP/mLuc, respectively. A) Experimental design: The schematic illustrates the study design for evaluating biodistribution and mRNA expression in B6129SF1/J mice. Mice received a single IV dose of the formulated LNPs, followed by intraperitoneal injection of D‐Luciferin after 24 h. Mice were then sacrificed, and major organs (heart, lungs, kidneys, spleen, and liver) were collected for bioluminescent and fluorescence imaging using the IVIS system. B) Biodistribution analysis: DiR fluorescence imaging and quantification of major organs revealed that the LNPs primarily accumulated in the liver and spleen, confirming their targeting efficiency. C) mRNA expression analysis: Bioluminescent IVIS imaging and quantification of luciferase expression in collected organs, at 24 h post‐injection, demonstrate that mLuc expression was predominantly localized to the spleen. Importantly, co‐delivery of 3M‐052 did not affect mLuc expression, suggesting that immune‐stimulatory modifications did not compromise mRNA translation. Further insights into the impact of LNP composition on biodistribution and mRNA expression are discussed in Figure S2 (Supporting Information).

To evaluate the ability of the LNP platform to target the spleen for mRNA expression, we developed DiR‐labeled fluorescent LNPs encapsulating firefly luciferase mRNA (mLuc). Two formulations were tested: one without 3M‐052 (DiR‐LNP/mLuc) and one containing 4.5% 3M‐052 (DiR‐3M‐LNP/mLuc) (**Figure** **3**A). Healthy B6129SF1/J mice (*n* = 3) were intravenously (IV) injected with DiR‐LNP/mLuc or DiR‐3M‐LNP/mLuc to deliver 0.3 mg kg^−1^ mLuc and 0.4 mg kg^−1^ 3M‐052. Mice received an intraperitoneal (IP) injection of D‐Luciferin 24 h after administration, followed by major organ collection for IVIS imaging to assess DiR fluorescence and luciferase bioluminescence (Figure 3A). The DiR fluorescence intensity was used to quantify the biodistribution of LNPs. The fluorescent imaging results demonstrated that most LNPs localized in the spleen and liver in both DiR‐LNP/mLuc and DiR‐3M‐LNP/mLuc treated animals (Figure 3B). No significant differences in organ distribution were observed between the two groups, indicating that 4.5% 3M‐052 did not affect LNP biodistribution. Luciferase expression intensity was then measured to quantify mRNA expression from the LNPs. The luciferase bioluminescent IVIS imaging results demonstrated that almost all luciferase expression (≥90%) occurred in the spleen for both DiR‐LNP/mLuc and DiR‐3M‐LNP/mLuc groups (Figure 3C). There was no significant difference in luciferase expression between these two groups, confirming that 4.5% 3M‐052 did not affect the mRNA expression of LNPs.

Figure S2 (Supporting Information) provides insight into the findings of Figure 3, clarifying why mRNA expression was primarily observed in the spleen despite nearly half of the nanoparticles accumulating in the liver. Previous studies have shown that PE‐based lipids, such as DOPE, exhibit a high affinity for complement component C3 in the protein corona, thereby enhancing C3 binding and facilitating APC uptake in the spleen.^[^ ^19^ ^]^ This mechanism promotes spleen‐targeting and has been leveraged by Harashima's lab to develop LNPs that enhance gene expression and immune activation.^[^ ^18^, ^20^ ^]^ Similarly, Shen's lab has demonstrated that lipid‐polymer nanoparticles designed for mRNA delivery selectively adsorb complement C3, further promoting spleen‐specific accumulation.^[^ ^21^ ^]^ In contrast, LNPs designed for liver targeting typically incorporate ionizable or permanently cationic lipids such as DLin‐KC2‐DMA, DLin‐MC3‐DMA, or ALC‐0315, which preferentially bind apolipoprotein E (ApoE).^[^ ^22^, ^23^ ^]^ This interaction facilitates hepatocyte uptake via LDL receptor‐mediated endocytosis. However, the liver's high enzymatic activity and metabolic turnover contribute to rapid mRNA degradation, limiting its potential for translation. By contrast, spleen‐resident dendritic cells provide a more favorable environment for endosomal escape and cytoplasmic release of mRNA, supporting enhanced gene expression.

### 2.2 Vaccination Using LNP/mKRAS with Co‐Encapsulated 3M‐052 Induces a Cytotoxic T Cell Response in an Orthotopic KPC Tumor Model

**Figure 4:**
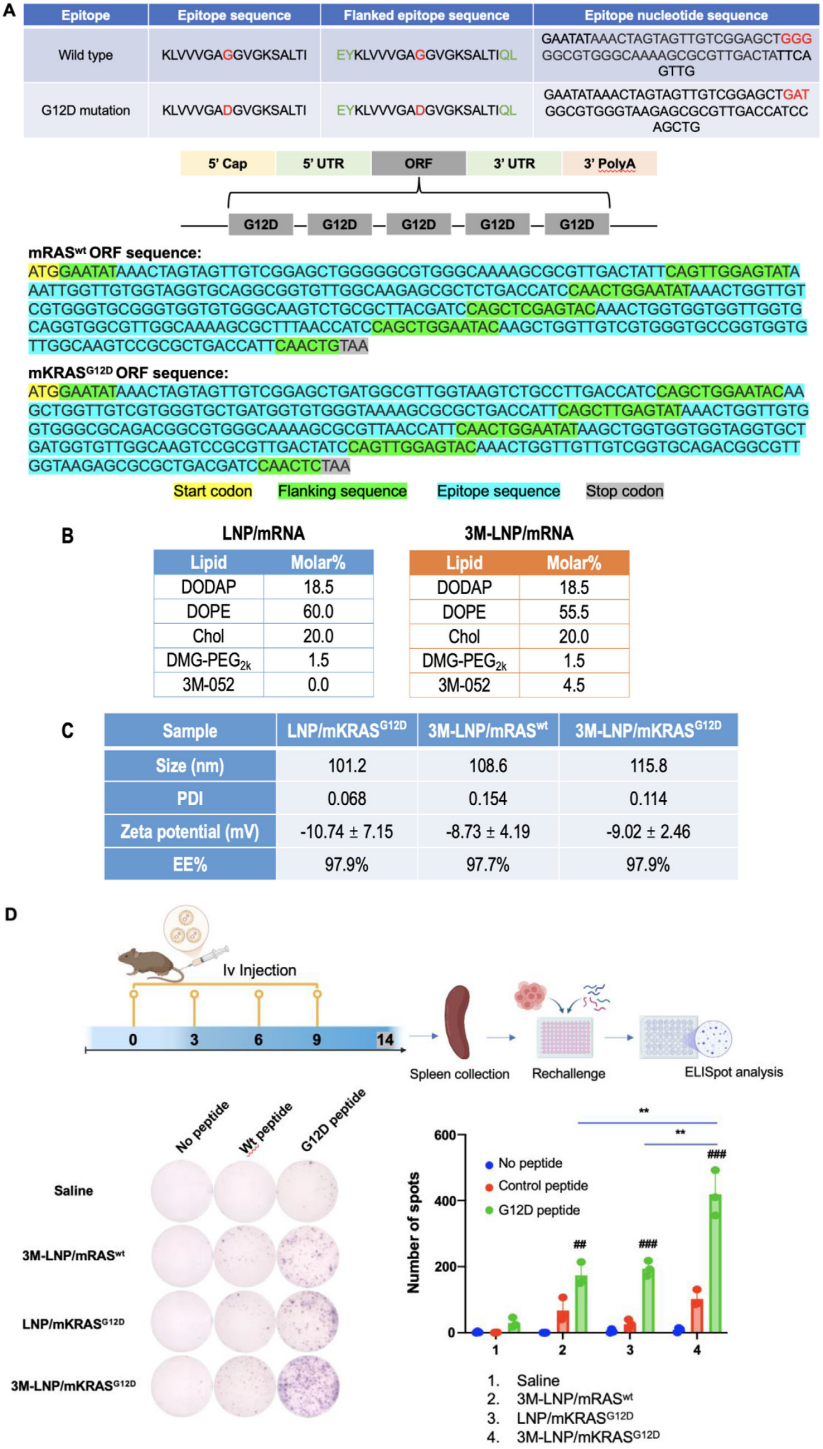
Design and evaluation of the spleen‐targeting LNP platform for IFN‐γ ELISPOT generation in B6129SF1/J mice. A) mRNA construct design: The epitope sequences of wild‐type RAS and the G12D‐mutated KRAS are depicted. The mRNA construct features a 5‐tandem‐repeat nucleotide sequence of RAS_5‐21_, flanked by two upstream and downstream amino acids. The ORF consists of a start codon, the epitope nucleotide sequence, and a stop codon, integrated with a 5′ Cap 1 structure, a 5′ UTR, the ORF, a 3′ UTR, and a Poly‐A tail at the 3′ end, forming the complete mRNA sequence. B) Lipid composition of LNP/mRNA formulations: the table summarizes the molar % of lipid components in LNP/mRNA and 3M‐LNP/mRNA formulations, detailing the structural differences between the standard and immune‐stimulatory LNPs. C) Characterization of spleen‐targeting LNPs: Key physicochemical properties of LNP/mKRAS^G12D^, 3M‐LNP/mRAS^wt^, and 3M‐LNP/mKRAS^G12D^ formulations are presented, including size, PDI, zeta potential and EE%. D) Experimental design, performance of ELISPOT analysis: Healthy mice received IV injections of different LNP formulations at designated time points. On Day 14, spleens were collected, and splenocytes were rechallenged with either a wild‐type peptide, G12D‐mutant peptide, or no peptide before analysis via an ELISPOT assay. Representative ELISPOT images demonstrate IFN‐γ release by T cells from the different treatment groups, including saline, 3M‐LNP/mRAS^wt^, LNP/mKRAS^G12D^, and 3M‐LNP/mKRAS^G12D^. The accompanying bar graph quantifies the number of IFN‐γ spots per group in response to different peptide stimulations.

KRAS frequently presents driver mutations in PDAC where G12 mutations dominate (≈99%), with KRAS G12D comprising up to 51%.^[^ ^24^ ^]^ We have previously demonstrated that the nanocarrier platforms for delivering KRAS^G12D^ mutant peptides and KRAS^G12D^ encoding mRNA could be successfully constructed for PDAC immunotherapy.^[^ ^5^, ^25^ ^]^ Consequently, we hypothesized that the spleen‐targeting LNP platform could deliver KRAS^G12D^ mRNA to APC in the spleen, thereby eliciting an efficient CTL response and enhancing the exogenous vaccination response through TLR7/8 pathway activation. The epitope sequences for both the wild‐type RAS and the G12D mutation are shown in **Figure** **4**A. These epitopes were paired with natural flanking sequences. Five tandem‐repeat epitope sequences were incorporated into an open reading frame (ORF). The resulting mRNA constructs were designed with a 5′ Cap 1 structure, a 5′ untranslated region (5′ UTR), the ORF, a 3′ UTR, and a Poly‐A tail at the 3′ end. These RNA strands were synthesized by TriLink Biotechnologies.^[^ ^5^ ^]^ All uridine nucleotides were replaced with N1‐methyl‐pseudouridine prior to purification. The gel analysis of the mRNA constructs provided by TriLink Biotechnologies is shown in Figure S3 (Supporting Information).

These epitope mRNA constructs were used to synthesize LNP/mKRAS^G12D^_,_ 3M‐LNP/mRAS^wt^, and 3M‐LNP/mKRAS^G12D^ using the same strategy as described in Figure 2B. The detailed lipid compositions of the particles are shown in Figure 4B, resulting in particles with a size range of 101.2 to 115.8 nm and polydispersity indexes (PDI) below 0.2. The encapsulation efficiency exceeded 97%, indicating that nearly all the mRAS^wt^ and mKRAS^G12D^ were successfully encapsulated within the LNP platforms (Figure 4C). These LNPs were selected for performing subsequent animal experiments.

To evaluate the capacity of 3M‐LNP/mRAS^wt^, LNP/mKRAS^G12D^, and 3M‐LNP/mKRAS^G12D^ to generate IFN‐γ‐producing T cells in the spleen, ELISPOT analysis was performed in healthy B6129SF1/J mice (*n* = 3), receiving IV particle injections as demonstrated in Figure 4D. Thus, these mice received either saline or the LNP injections delivering 1.25 mg kg^−1^ mRNA or 1.75 mg kg^−1^ 3M‐052 every 3 days for a total of 4 administrations (Figure 4D). On day 14, mice were sacrificed, and splenocytes were harvested to perform IFN‐γ ELISPOT assays on splenocytes stimulated with the mutant KRAS or wildtype RAS peptides (Figure 4D). While splenocytes from the saline control group showed almost no IFN‐γ producing colonies in response to exposure to either peptide, splenocytes from LNP‐treated mice demonstrated significant IFN‐γ spot formation in response to G12D, compared to a weak response to wildtype peptide. Notably, 3M‐LNP/mKRAS^G12D^ induced the most robust effect, which was statistically significant (*p* < 0.05) compared to both 3M‐LNP/mRAS^wt^ and LNP/mKRAS^G12D^.

To develop an orthotopic KPC tumor model that closely mimics the characteristics of human PDAC, we used KRAS‐transformed murine pancreatic adenocarcinoma cells with stable luciferase expression (KPC‐luc cells), derived from a spontaneous tumor in a transgenic KrasLSL‐G12D/+; Trp53LSL‐R172H/+; Pdx‐1‐Cre mouse model.^[^ ^6^, ^7^, ^8^, ^9^ ^]^

**Figure 5:**
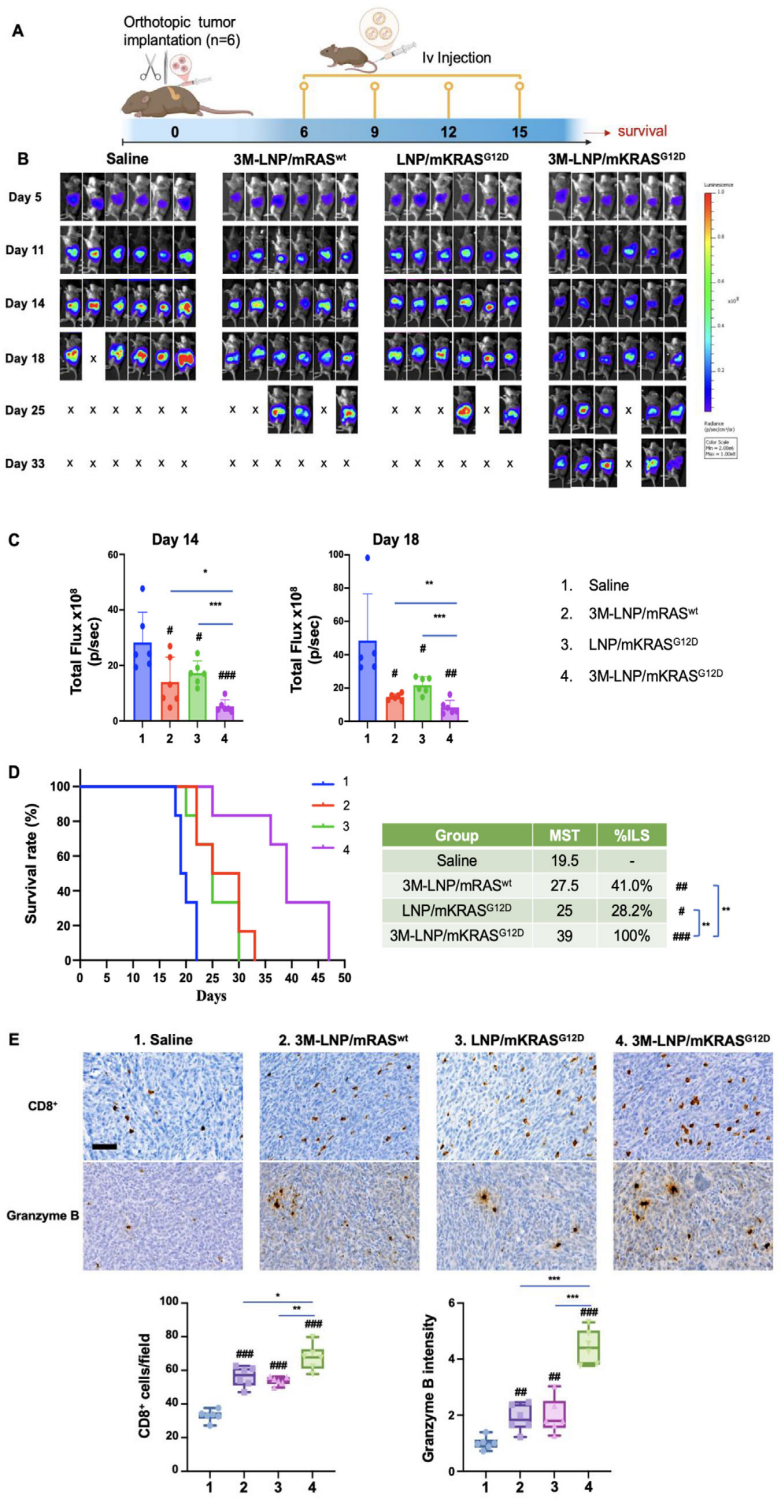
Evaluation of immune responses induced by the spleen‐targeting LNP System. A) Experimental design: Orthotopic KPC‐luc tumor cells were implanted into the pancreatic tails of B6129SF1/J mice. Tumor‐bearing mice were randomly assigned to 4 treatment groups (*n* = 6): saline, 3M‐LNP/mRAS^wt^, LNP/mKRAS^G12D^, and 3M‐LNP/mKRAS^G12D^. Each group received IV injections every 3 days for a total of 4 doses (mRNA at 1.25 mg kg^−1^; 3M‐052 at 1.75 mg kg^−1^). Mice were monitored daily until they met euthanasia criteria or expired naturally. B) Tumor imaging: In vivo IVIS) was utilized to monitor orthotopic tumor progression across all treatment groups at specified time points. C) Tumor bioluminescence quantification: Tumor bioluminescence was measured on Days 14 and 18 post‐treatment initiation to assess tumor burden. D) Survival analysis: Kaplan–Meier survival curves were generated to evaluate the efficacy of each treatment regimen. MST and %ILS are detailed in the accompanying table. Data are presented as mean ± SEM. Statistical significance is denoted as follows: #*p* < 0.05; ##*p* < 0.01; ###*p* < 0.001; *p < 0.05; **p < 0.01; ***p < 0.001. E) IHC Analysis: Tumor tissues were subjected to IHC staining to detect CD8⁺ T cell infiltration and Granzyme B expression. Representative images and quantification data indicate the extent of immune cell infiltration and cytotoxic activity within tumor tissues across different treatment groups. Five random fields per sample were analyzed. Scale bar represents 50 µm.

Orthotopic tumor‐bearing B6129SF1/J female mice were established by injecting KPC‐luc cells into the pancreas tail by a short survival surgery procedure (**Figure** **5**A). After confirming tumor growth by IVIS imaging, the tumor‐bearing mice (*n* = 6) received IV injections every 3 days for a total of 4 administrations with either saline, 3M‐LNP/mRAS^wt^, LNP/mKRAS^G12D^, and 3M‐LNP/mKRAS^G12D^ (mRNA, 1.25 mg kg^−1^; 3M‐052, 1.75 mg kg^−1^). IVIS imaging was performed on days 5, 11, 14, 18, 25, and 33, as displayed in Figure 5B. Quantitative analysis of tumor bioluminescence intensity on days 14 and 18 demonstrated a significant luminescence reduction in mice treated with 3M‐LNP/mRAS^wt^, LNP/mKRAS^G12D^, and 3M‐LNP/mKRAS^G12D^ compared to the saline control, emphasizing the efficacy of encapsulated 3M‐052 and mKRAS^G12D^ delivery (Figure 5C). Notably, the best outcome was achieved with 3M‐LNP/mKRAS^G12D^, indicating a significant synergistic effect from the dual delivery spleen‐targeting LNPs.

Animals were provided with supportive care and monitored regularly until they met euthanasia criteria or expired naturally according to UCLA Animal Research Committee (ARC) regulations, allowing survival analysis by plotting Kaplan–Meier curves. The results demonstrated a significant survival improvement in animals treated with the LNP formulations compared to saline (Figure 5D). While the median survival time (MST) of 3M‐LNP/mRAS^wt^ (*p* < 0.01) and LNP/mKRAS^G12D^ (*p* < 0.05) was significantly extended compared to the saline control, the best survival was obtained with the 3M‐LNP/mKRAS^G12D^ (*p* < 0.001). The MST of 39 days and a 100% increase in life span survival (%ILS) in the 3M‐LNP/mKRAS^G12D^ group were significantly better than single encapsulation groups.

To confirm activation of CTL in tumor cell killing, immunohistochemistry (IHC) analysis for CD8⁺ T cell infiltration and granzyme B expression was performed on primary tumor tissues (Figure 5E). While both 3M‐LNP/mRAS^wt^ and LNP/mKRAS^G12D^ treatment could be seen to increase CD8⁺ T cell expression at the tumor site compared to the saline control, the administration of 3M‐LNP/mKRAS^G12D^ solicited the highest cell density, which was significantly different from the other treatment groups (*p* < 0.001). These CTLs are recruited from the spleen by a mechanism involving CXCR3 and CXCL10.^[^ ^26^, ^27^, ^28^, ^29^, ^30^ ^]^ Similarly, while all LNP treatments enhanced granzyme B deposition, the results of 3M‐LNP/mKRAS^G12D^ was significantly different from the other treatment groups (*p* < 0.001), highlighting the adjuvant effect of 3M‐052.

All considered, the data in Figures 4 and 5 demonstrate that the delivery of mKRAS^G12D^ mRNA to the spleen can successfully trigger an exogenous vaccination response, which was further boosted by co‐encapsulation of a TLR7/8 agonist, resulting in strengthening of the PDAC immunity cycle.

### 2.3 The Combination of Exogenous LNP Vaccination with Chemo‐Immunotherapy Augments the PDAC Immunity Cycle, and Survival Outcome

A major challenge in treating PDAC is dismantling its immunosuppressive microenvironment to elicit a potent, durable immune response. To address this, we have previously developed a silicasome‐based irinotecan delivery platform that not only enhances drug delivery but also induces ICD (Figure S4, Supporting Information).^[^ ^6^, ^7^, ^31^, ^32^ ^]^ As illustrated in **Figure** **6**A (left panel), irinotecan induces (ICD by exposing CRT as an “eat me” signal while releasing HMGB1 and ATP, which serve as DAMPs that induce the activation and maturation of APC.^[^ ^3^, ^6^, ^25^ ^]^ This process primes CTL against tumor antigens, with the support of the spleen and regional lymph nodes, effectively generating an “endogenous vaccination” response. However, due to the low neoantigen burden in pancreatic ductal PDAC, this response may be insufficient to sustain a robust immune activation. To enhance this effect, we incorporated a TLR7/8 agonist in both nanocarriers used for experimentation in Figure 6. We also hypothesized that an “exogenous vaccination” strategy could further reinforce the PDAC immunity cycle by delivering mutant KRAS mRNA to the spleen. This approach aims to amplify tumor‐specific T cell activation, complementing the endogenous immune response. The right panel of Figure 6A illustrates the proposed synergistic contribution of the exogenous vaccination strategy, acting to strengthen the immunity cycle by improved antigen presentation in the spleen.

Figure 6Synergistic effect of combined vaccination strategies in orthotopic KPC tumor model. A) Schematic overview of vaccination responses: The left panel illustrates endogenous vaccination via silicasome‐delivered irinotecan, which inhibits topoisomerase I, inducing ER stress and ICD. This process releases CRT and DAMPs like HMGB1 and ATP; CRT serves as an "eat‐me" signal, facilitating tumor cell uptake, while DAMPs promote APC maturation. The right panel depicts exogenous vaccination through 3M‐LNP/mKRAS^G12D^, delivering KRAS^G12D^ mRNA and the TLR7/8 agonist 3M‐052 to splenic APC. This involves endocytosis, TLR7/8 activation, mRNA endosomal escape, translation, MHC epitope processing, and presentation, assisting CTL activation. As detailed in Figure 1, enhancing splenic APC activation and CTL recruitment is hypothesized to bolster the ICD‐mediated endogenous vaccination response. B) Experimental design: Orthotopic KPC‐luc tumor cells were implanted into the pancreatic tails of B6129SF1/J mice (*n* = 4). Mice received intravenous injections of saline, 3M‐Si‐IR, 3M‐LNP/mKRAS^G12D^, or a combination of 3M‐Si‐IR and 3M‐LNP/mKRAS^G12D^, administered twice weekly for two weeks. C) Tumor growth assessment: IVIS images display tumor progression across treatment groups. Quantification of tumor bioluminescence on Days 12 and 18 is provided. D, E) IHC analysis: Representative IHC images and quantification reveal CD8⁺ T cell infiltration, Granzyme B expression, CRT intensity, IFN‐γ intensity and PD‐1 expression in primary tumors from each treatment group. Five fields were randomly selected for analysis. Scale bar: 50 µm. Data represent mean ± SEM. Statistical significance is annotated as follows: #*p* < 0.05; ##*p* < 0.01; ###*p* < 0.001; *p < 0.05; **p < 0.01; ***p < 0.001.

**Figure:**
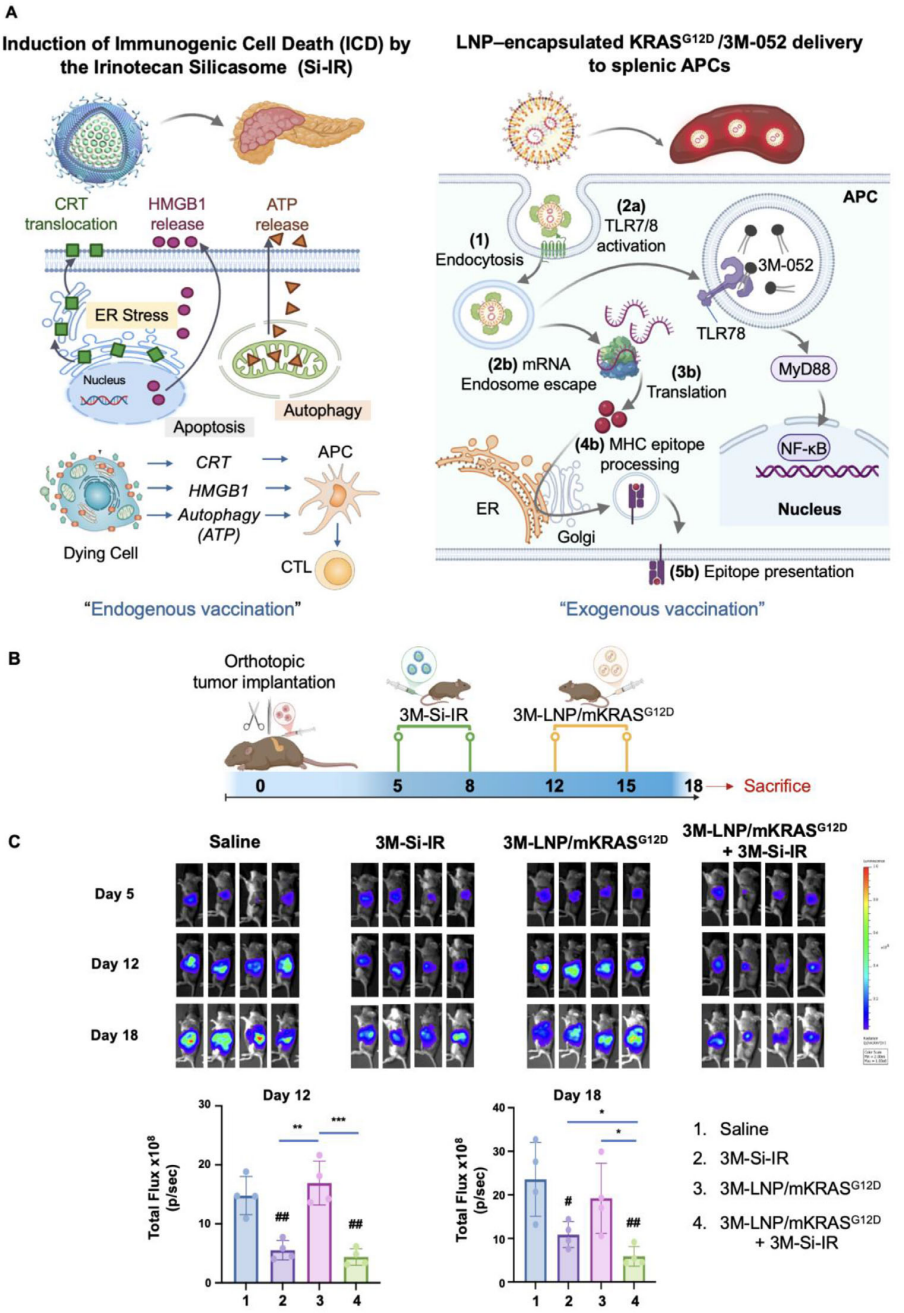

**Figure:**
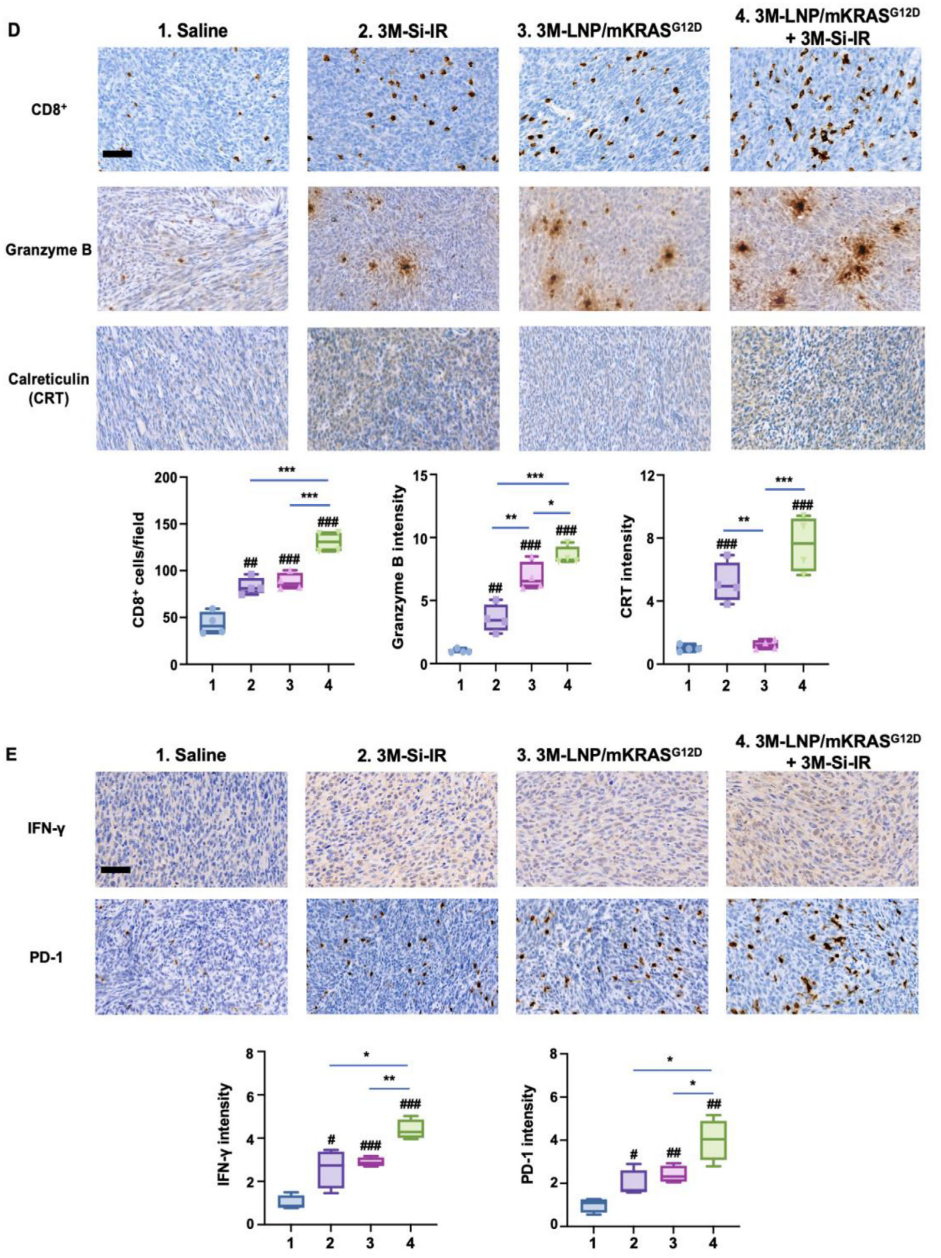

To test our hypothesis, we synthesized a fresh batch of 3M‐LNP/mKRAS^G12D^ alongside MSNPs to form silicasomes, as previously described by us.^[^ ^6^, ^7^, ^9^ ^]^ Online Figure S4 (Supporting Information) explains the historical use of the silicasomes, including how their coated lipid bilayer facilitates remote loading of irinotecan using a trapping agent. It also illustrates how the 3M‐052 lipid tail can be efficiently incorporated into the coated bilayer, resulting in dual drug delivery silicasomes (designated 3M‐Si‐IR), capable of improving the pharmacokinetics of irinotecan and 3M‐052 delivery to the orthotopic PDAC site, using a transcytosis process (Figure S4, Supporting Information).^[^ ^7^, ^32^ ^]^ Figure S5 (Supporting Information) presents the physicochemical characterization of the newly synthesized 3M‐Si‐IR particle batch used for the current studies.

Orthotopic KPC tumors were established in animals (*n* = 4) and treated with biweekly IV injections on days 5 and 8, receiving either saline or 3M‐Si‐IR (3M‐052, 2 mg kg^−1^; irinotecan, 40 mg kg^−1^), followed by additional biweekly injections on days 12 and 15 with either saline or 3M‐LNP/mKRAS^G12D^ (mKRAS^G12D^, 1.25 mg kg^−1^; 3M‐052, 1.75 mg kg^−1^) (Figure 6B). In contrast to survival studies where 4 doses of 3M‐LNP/mKRAS^G12D^ were administered over a prolonged period, the mechanistic study depicted in Figure 6B was limited to 2 doses due to the earlier tissue collection time point (day 18), selected to ensure analysis of immune responses in viable animals. Tumor progression was monitored via IVIS imaging on days 5, 12, and 18 (Figure 6C). Quantitative analysis of tumor bioluminescence revealed a significant reduction in luminescence intensity in mice treated with 3M‐Si‐IR, with or without 3M‐LNP/mKRAS^G12D^, compared to saline or 3M‐LNP/mKRAS^G12D^ alone. Moreover, the combination of silicasomes and LNPs resulted in a statistically significant tumor reduction beyond that observed with monotherapy, highlighting the synergistic impact of a combined endogenous and exogenous vaccination strategy.

On day 18, following animal sacrifice, the primary tumors were harvested and partitioned for IHC and transcriptomic analysis. IHC staining was performed to assess the expression of CD8⁺ T cells, granzyme B, and CRT at the tumor site. Visual inspection showed increased staining intensity for all three markers in tumors treated with 3M‐Si‐IR and 3M‐LNP/mKRAS^G12D^, with the combination therapy providing the most prominent increase (Figure 6D, upper panel). Quantitative analysis across multiple fields of view (4 animals per group, 5 fields per animal) confirmed statistically significant increases in all 3 parameters (Figure 6D, lower panel). Both 3M‐Si‐IR and 3M‐LNP/mKRAS^G12D^ significantly enhanced CD8⁺ T cell infiltration and granzyme B expression compared to saline (*p* < 0.01, p < 0.001, respectively). Notably, combination treatment induced the highest CD8⁺ T cell density and CTL activity (*p* < 0.001). Furthermore, CRT expression, a marker of ICD, was significantly elevated in the 3M‐Si‐IR and combination groups compared to saline (*p* < 0.001) and 3M‐LNP/mKRAS^G12D^ alone (*p* < 0.01, *p* < 0.001). This confirmed our previous data (Figure S4F, Supporting Information). In response to recent insights into T cell activation and exhaustion dynamics, we also expanded our analysis to further characterize the functional status of tumor‐infiltrating lymphocytes following combination therapy. IHC staining revealed increased expression of IFN‐γ and PD‐1 within tumors, indicative of active T cell engagement alongside the emergence of checkpoint‐mediated regulation (Figure 6E, upper panel). Quantified data demonstrate a significant upregulation of IFN‐γ expression in the 3M‐Si‐IR, 3M‐LNP/mKRAS^G12D^ and combination groups compared to saline (*p* < 0.01, *p* < 0.001, *p* < 0.001, respectively), with the combination treatment displaying the highest IFN‐γ expression (*p* < 0.05 vs 3M‐Si‐IR, *p* < 0.01 vs 3M‐LNP/mKRAS^G12D^). Similarly, PD‐1 expression was significantly increased in all treatment groups compared to saline (*p* < 0.05, *p* < 0.01, *p* < 0.01, respectively), with combination treatment exhibiting the most pronounced PD‐1 upregulation ((*p* < 0.05 vs both monotherapies). These findings indicate that combination treatment induces potent CTL activation while also engaging immune checkpoint feedback mechanisms in the tumor microenvironment.

**Figure 7:**
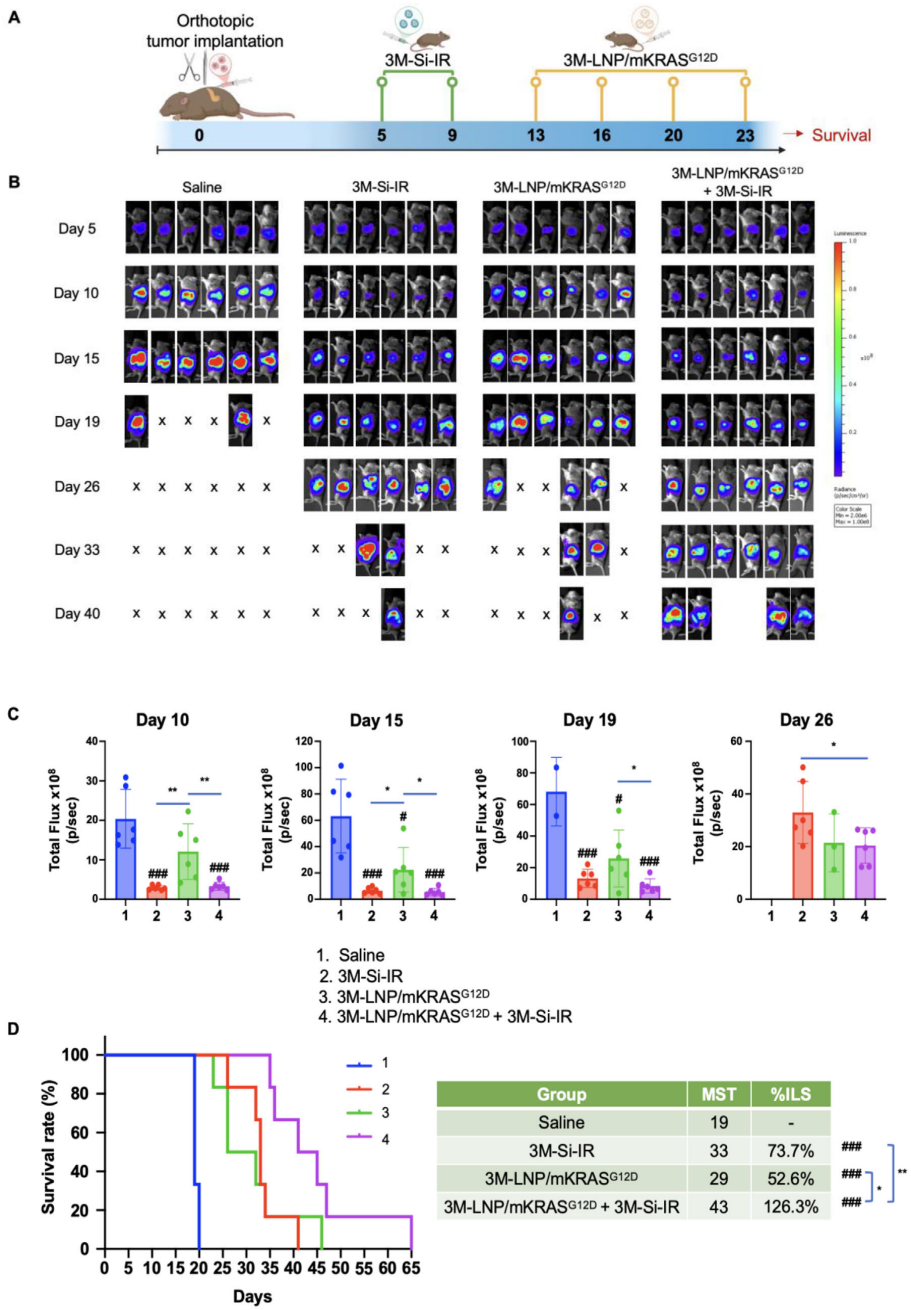
Combination of 3M‐LNP/mKRAS^G12D^ with 3M‐Si‐IR prolong survival in orthotopic tumors. A) Experimental outline of the study conducted in an orthotopic KPC tumor model, looking at the survival impact of triggering the endogenous ICD pathway before administration of the exogenous LNP vaccine. B) IVIS imaging was performed at multiple time points. C) Quantification of tumor bioluminescence intensity for each group on days 10, 15, 19, and 26. Significant tumor reduction was observed with combination therapy. D) Kaplan–Meier plots to display the different animal groups' survival rates, MST, and %ILS. Data represent mean ± SEM. #*p* < 0.05; ###*p* < 0.001; *p < 0.05; **p < 0.01.

Building on the experiment in Figure 6, we next investigated whether combining endogenous and exogenous vaccination would enhance survival in an orthotopic KPC tumor model. Mice (*n* = 6) were randomly assigned to receive two IV injections of either saline or 3M‐Si‐IR (3M‐052, 2 mg kg^−1^; irinotecan, 40 mg kg^−1^), followed by 4 IV injections of either saline or 3M‐LNP/mKRAS^G12D^ (mKRAS^G12D^, 1.25 mg kg^−1^; 3M‐052, 1.75 mg kg^−1^) (**Figure** **7**A). Tumor progression was monitored via IVIS imaging (Figure 7B), and animals were provided with supportive care and monitored regularly until they met euthanasia criteria or expired naturally according to UCLA ARC regulations.

Quantitative analysis of tumor bioluminescence on days 10, 15, 19, and 26 (Figure 7C) showed that combination therapy (3M‐Si‐IR + 3M‐LNP/mKRAS^G12D^) produced the greatest tumor growth inhibition compared to saline and either monotherapy, in agreement with the survival improvement. Although both 3M‐Si‐IR (p < 0.001) and 3M‐LNP/mKRAS^G12D^ (*p* < 0.001) monotherapies extended survival relative to saline, the combination therapy yielded the highest MST of 43 days and a 126.3% increase in lifespan (*p* < 0.001) (Figure 7D). These findings reinforce our hypothesis that integrating endogenous and exogenous vaccination strategies synergistically augments the anti‐PDAC immune response, strengthening the PDAC immunity cycle.

The experiments in Figures 6 and 7 were repeated with partial reversal of the order of administration of the exogenous and endogenous stimuli, as shown in online Figure S6 (Supporting Information). The results confirmed that pre‐treatment with 3M‐LNP/mKRAS^G12D^ followed by Si‐IR with coupled LNP/Si‐IR administration did not alter overall outcome. Collectively, these findings highlight that combination therapy with silicasome and LNP carriers enhances the anti‐PDAC immune response. The observed synergistic effect underscores the need for further investigation to elucidate the mechanistic basis of synergy ‐ specifically, the interaction between ICD induction by silicasomes and the impact of splenic targeting by LNPs. To more comprehensively explore this, without reverting to additional histochemistry or flow cytometry procedures, we performed bulk RNA sequencing (RNA‐Seq) on tumor tissues, analyzing gene sets related to ICD, antigen presentation, APC activation, and T cell activation, as described in the next section.

### 2.4 Transcriptomic RNA Analysis Unravels the Synergistic Effects of Nanocarrier‐Induced ICD and Spleen‐Targeted APC Activation

To more extensively elucidate the immune mechanisms underlying irinotecan‐induced ICD and the antigen presentation driven by 3M‐LNP/mKRAS^G12D^ ‐ both critical components of the cancer immunity cycle ‐ we performed bulk RNA sequencing on primary PDAC tissues collected from the experiment performed in Figure 6. Tumors were preserved in RNAlater buffer, and total RNA was extracted for mRNA enrichment, fragmentation, cDNA synthesis, and amplification. High‐throughput sequencing and data analysis were conducted for gene feature annotation and generating immune profiles reflecting ICD responses and antigen presentation pathways leading to T cell activation (**Figure** **8**A).

Figure 8Transcriptomic analysis of immune activation by combing endogenous and exogenous vaccination in the primary tumor microenvironment. A) Schematic representation of the transcriptomic analysis approach. Primary tumors from Figure 6 were dissected and total RNA was extracted, followed by separation, enrichment, fragmentation, reverse transcribed into cDNA and amplification for sequencing. Following sequencing and alignment, raw read counts from STAR were normalized using the MRN method to correct for sequencing depth variations. To assess the impact of irinotecan‐induced ICD at the tumor site and spleen‐targeted KRAS delivery via LNPs, gene sets associated with apoptosis, ER stress, DAMP release, APC activation, cytokine secretion, and antigen presentation were extracted from the KEGG pathway database. To compare relative expression patterns across treatment groups, z‐score transformation was applied to normalize gene expression values. B) Heatmap analysis of 21 key genes associated with treatment‐induced immune responses, categorized into six groups: apoptosis, ER stress/ICD, APC activation, T cell activation, T cell recruitment, and CTL killing. C) Heatmap of gene expression of translation products playing a role in antigen processing in the MHC I pathway. Tumor antigens are processed via the proteasome, transported by TAP, and loaded onto MHC I molecules, facilitating recognition by CD8⁺ T cells. D) Heatmap of gene expression of translation products associated with antigen processing MHC II pathway. Tumor antigens are processed in endosomes, loaded onto MHC II molecules, and presented to CD4⁺ helper T cells to stimulate an immune response.

**Figure:**
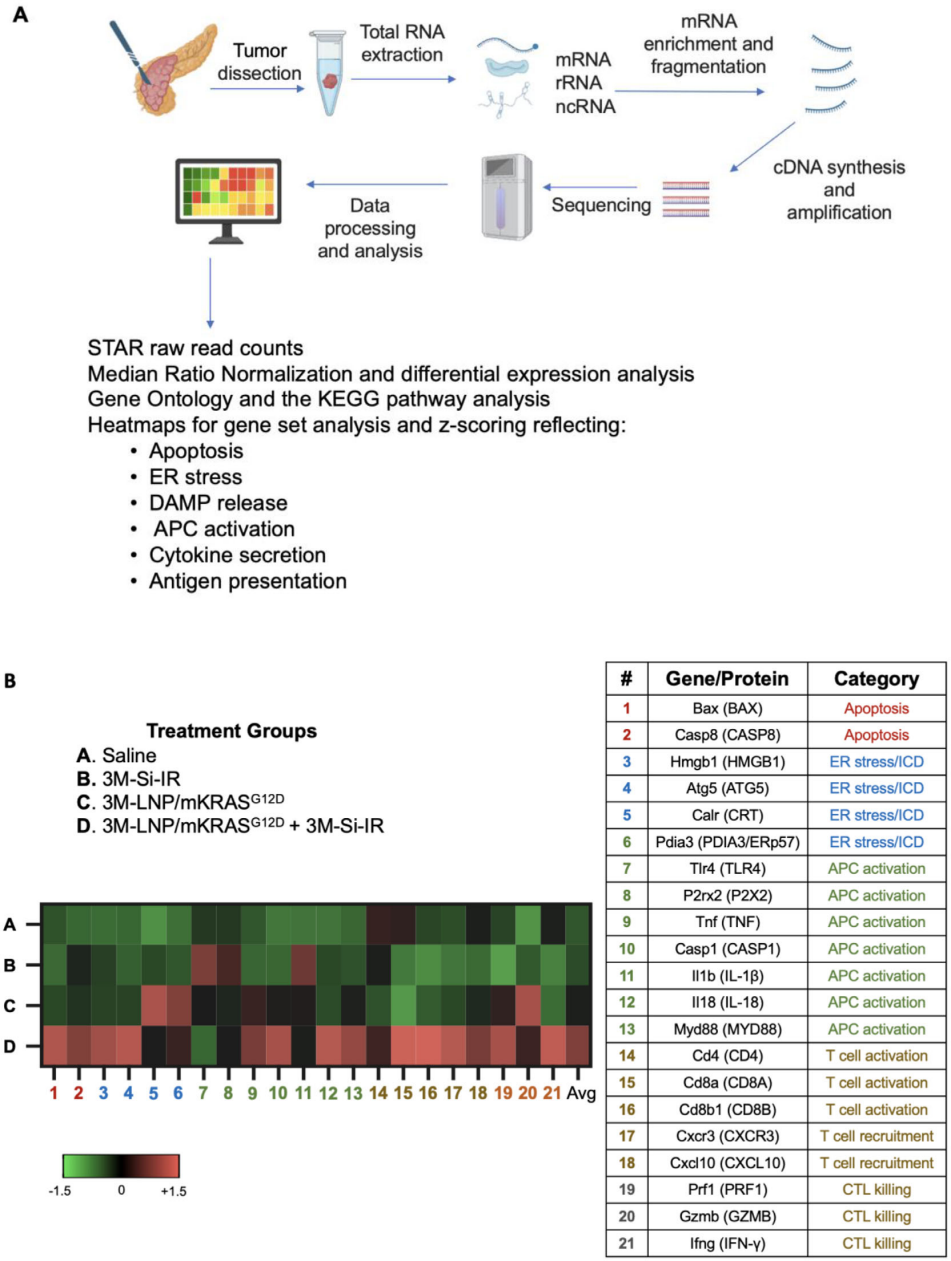

**Figure:**
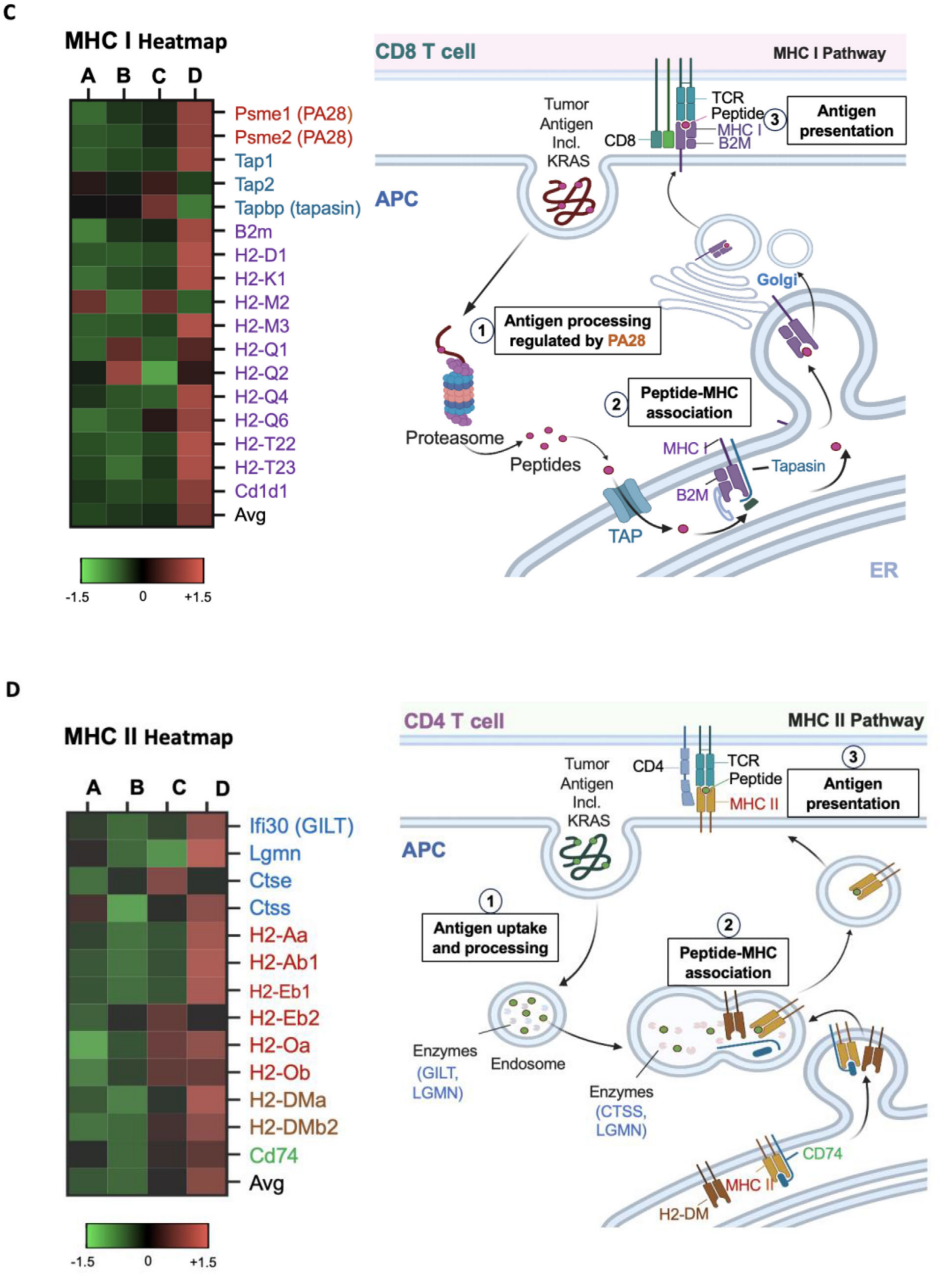

After sequencing and alignment, raw read counts obtained from STAR were normalized using the Median Ratio Normalization (MRN) method to correct for variations in sequencing depth. To evaluate the effects of irinotecan‐induced ICD at the tumor site and splenic KRAS delivery by LNPs, gene sets that reflect apoptosis, ER stress, DAMP release, APC activation, T cell activation, cytokine secretion, and antigen presentation were extracted from Gene Ontology (GO) and KEGG pathway databases. To compare relative gene expression across treatment groups, z‐score transformation was applied to the normalized data and presented as a heatmap in Figure 8B, which illustrates the expression of 21 key genes in the following treatment comparison groups: saline (Group A), 3M‐Si‐IR (Group B), 3M‐LNP/mKRAS^G12D^ (Group C), and combination therapy (Group D). The most pronounced effects were observed in Group D, where combination therapy led to greater immune activation compared to saline and monotherapy groups.

The heatmap in Figure 8B captures specific gene expression changes across several functional categories, including: i) Apoptosis induction: Upregulation of BAX and caspase‐8, predominantly in response to combination therapy; ii) ICD markers: Irinotecan‐induced ER stress, accompanied by increased expression of HMGB1 and Atg5 (an autophagy marker linked to ATP release), acting as DAMP molecules involved in APC activation and maturation. The ER chaperone ERp57 complexes to CRT, further amplifying the ICD response; iii) APC activation & cytokine release: TLR4 upregulation is involved in triggering MyD88‐dependent signaling in response to HMGB1, supporting the production of pro‐inflammatory cytokines such as TNF‐α. Additionally, P2RX7 interacts with extracellular ATP, driving NLRP3 inflammasome activation and caspase‐1‐mediated maturation of IL‐1β and IL‐18; iv) T cell activation, differentiation, recruitment and cytotoxic killing: Upregulation of Cd4, Cd8a, and Cd8b1 highlights increased expression of co‐stimulatory surface receptors on CD4⁺ and CD8⁺ T cells. Elevated expression of Cxcr3 and Cxcl10 is compatible with chemokines mediating enhanced CTL recruitment from secondary lymphoid tissues to the tumor site. Increased levels of IFN‐γ, perforin, and granzyme B indicate heightened CTL cytotoxicity directed against the tumor. Additional transcriptomics analyses reflecting T cell activation, T cell exhaustion and transcription factor expression appear in Figures S7–S9 (Supporting Information). Notably, Figure S9 (Supporting Information) shows increased expression of the transcription factor BATF (Basic Leucine Zipper ATF‐Like Transcription Factor), which plays a key role in promoting the effector and memory differentiation of helper T (CD4⁺) cells, particularly within the context of immune activation and response. Overall, these findings suggest that combination therapy enhances immune activation, APC maturation, and CTL recruitment, driving a stronger anti‐PDAC response through the combinatorial effects of endogenous and exogenous vaccination mechanisms.

Figure 8C presents heatmap analysis of key genes involved in MHC I‐mediated antigen presentation, illustrating the effects of treatment groups A‐D on neoantigen processing and display. The heatmap reveals a marked increase in the expression of MHC I pathway genes in the combination therapy group (3M‐LNP/mKRAS^G12D^ + 3M‐Si‐IR) compared to saline and monotherapy groups, indicating a more efficient antigen processing and presentation mechanism ‐ a crucial step for activating cytotoxic T lymphocytes and driving tumor elimination. As depicted in the accompanying diagram, upregulation of the proteasome activator 28 complex (Psme) reflects its central role in facilitating the degradation of tumor antigens into peptide fragments within the MHC I pathway. These antigenic peptides are then transported into the ER by the TAP heterodimer (TAP1 and TAP2), where they are loaded into the MHC I groove by tapasin, a component of the peptide‐loading complex. Once the peptide epitopes are successfully loaded, the MHC I complexes—that include H2 (D1, K1, M2, M3, Q1, Q2, Q4, Q6, T22, and T23) and Cd1d1 components—traffic to the cell surface, where they are presented to CD8^+^ T cells to initiate a targeted immune response.

In addition, antigen presentation through the MHC II pathway is illustrated by the heatmap and accompanying diagram in Figure 8D. Here, exogenous antigens are internalized via the endosomal route and degraded into peptides by enzymes such as gamma‐interferon‐inducible lysosomal thiol‐protease and legumain (encoded by GILT and LGMN). Immature MHC II complexes, stabilized by the invariant chain (CD74), are delivered to specialized endosomal compartments where this chain is involved in replacing the CLIP fragment with antigenic peptides, with the assistance of H2‐DMa and H2‐DMb2 molecules and enzymes like cathepsin S (encoded by CTSS) and legumain. These mature MHC II complexes, including H2‐Aa, H2‐Ab1, and H2‐Eb2, are transported to the cell surface for presentation to CD4⁺ T cells. The heatmap results show that the combination therapy group induced the highest expression of genes associated with the MHC II pathway, suggesting enhanced antigen presentation that not only promotes APC maturation for indirect CD8⁺ T cell priming but also supporting the MHC I pathway by activating CD4⁺ T cells to further stimulate CD8⁺ T cell proliferation and cytotoxic function.

Additional transcriptomic analyses, discussed in Supporting Information, demonstrate upregulation of cytotoxic markers (perforin) as well as immune checkpoint receptors (PD‐1, LAG‐3, TIGIT), consistent with a vigorous, yet regulated, T cell response (Figures S7 and S8, Supporting Information). Transcriptomic profiling of key transcription factors further supports this duality, showing increased expression of TOX (associated with exhaustion), T‐bet and BATF (associated with effector and memory T cell development) (Figure S9, Supporting Information). These findings underscore the rationale for future studies combining our strategy with immune checkpoint inhibitors to prevent T cell exhaustion and enhance durable tumor control.

Collectively, these gene expression findings in Figure 8, Figures S7–S9 (Supporting Information) verify that the cancer immunity cycle is effectively completed by priming with 3M‐Si‐IR‐mediated endogenous vaccination followed by boosting with 3M‐LNP/mKRAS^G12D^ as an exogenous vaccine.

## 3 Discussion

We hypothesized that effectively countering the immunosuppressive landscape of PDAC requires a multifaceted strategy ‐ one that not only induces ICD but also augments the cancer immunity cycle to sustain a durable antitumor response. Here, we introduce a novel combinatorial approach that harnesses the regional effects of irinotecan‐induced ICD with the systemic immune‐stimulating potential of KRAS^G12D^ mRNA delivery, co‐formulated with the TLR7/8 agonist, 3M‐052. To maximize both local and systemic immune activation, we engineered two specialized nanocarrier platforms. Silicasomes ‐ mesoporous silica nanoparticles enveloped in a lipid bilayer ‐ were utilized to efficiently deliver irinotecan to the tumor site, ensuring robust ICD induction and the subsequent release of tumor‐associated antigens and DAMPs. In parallel, we designed a cationic LNPs system optimized for spleen targeting via a DODAP/DOPE lipid composition, facilitating the delivery of KRAS^G12D^ mRNA and 3M‐052 to enhance antigen presentation and T cell priming.

Using an orthotopic KPC model of pancreatic cancer, we conducted a rigorous evaluation of this dual‐platform therapy, assessing its impact on tumor progression, survival, and immune activation. The combination therapy resulted in significantly enhanced tumor regression and prolonged survival compared to monotherapies alone. Bulk RNA sequencing and gene expression analysis provided mechanistic insights, revealing upregulation of key immune pathways, including apoptosis, endoplasmic reticulum stress, antigen presentation via MHC I and II, T cell activation, and T cell exhaustion. These findings underscore the potential of this strategy to bridge innate and adaptive immunity in the cancer immunity cycle, effectively overcoming the immunosuppressive barriers of PDAC and initiating a coordinated, self‐sustaining antitumor immune response.

One of the most significant findings of our study is the demonstration that the combination therapy elicits a potent immune response that far exceeds the effects of either monotherapy alone. Irinotecan‐loaded silicasomes efficiently induce ICD, triggering the release of DAMPs and “eat me” signals that enhance tumor antigen uptake by APC. This endogenous vaccination effect is further amplified by the spleen‐targeting cationic LNPs, which co‐deliver KRAS^G12D^ mRNA and the TLR7/8 agonist 3M‐052. This formulation drives robust APC activation in the spleen, catalyzing a systemic antitumor immune response. Effector T cells primed in the spleen by the combination therapy migrates to the tumor site via chemokine‐guided trafficking mechanisms involving CXCR3 and CXCL10 in the tumor microenvironment (Figure 8B). The contribution of this pathway has also previously been documented in successful T cell recruitment to PDAC and other solid tumor microenvironments.^[^ ^26^, ^27^, ^28^, ^29^, ^30^ ^]^ Bulk RNA sequencing corroborates these findings, revealing the integration of key molecular signatures of immune activation, including the upregulation of genes involved in apoptosis, ER stress, and antigen presentation through both MHC I and II pathways, and T cell recruitment (Figure 8). Notably, this combinatorial approach not only strengthens the innate immune response but also bridges into adaptive immunity, fostering enhanced CD8⁺ T cell priming and activation. The heightened expression of genes involved in antigen presentation reinforces our hypothesis that this dual‐platform strategy overcomes PDAC's formidable immune evasion mechanisms, ultimately transforming an otherwise immunosuppressive tumor microenvironment into one primed for sustained antitumor activity.

Inducing ICD has the potential of being a crucial element in successful cancer immunotherapy, as it enables dying tumor cells to become a source of endogenous antigens that stimulate the immune system.^[^ ^33^, ^34^ ^]^ Our findings confirm previous demonstration that irinotecan‐loaded silicasomes effectively trigger ICD, promoting the release of key DAMPs and upregulating tumor cell surface expression of CRT ‐ critical signals that enhance antigen uptake and maturation of dendritic cells (DCs).^[^ ^6^, ^7^ ^]^ This process mirrors the mechanism of exogenous vaccination, where antigen exposure drives immune system priming and activation. However, the effectiveness of ICD is contingent upon both the immunogenicity of the tumor and the participation of secondary lymphoid structures, such as the spleen.^[^ ^35^, ^36^ ^]^

While tumors with a high mutational burden, like melanoma or lung cancer, generate an abundance of neoantigens that facilitate immune recognition, PDAC is characterized by a lower mutational burden and an immunosuppressive TIME, both of which limit the efficacy of endogenous tumor antigens in generating a robust immune response.^[^ ^37^, ^38^, ^39^, ^40^, ^41^ ^]^ Furthermore, even when tumor neoantigens are present in PDAC, immune evasion mechanisms often hinder their processing and presentation by DCs, reinforcing the tumor's resistance to immunotherapy.^[^ ^3^, ^42^, ^43^ ^]^ Given these challenges, our approach of targeting the spleen with a KRAS vaccine presents a compelling strategy to enhance PDAC immunity by improving antigen presentation and T cell priming. However, it is also important to consider that the spleen is also a source of immunosuppressive cells, including myeloid derived suppressor cells (MDSC), regulatory T cells (Tregs), and M2 macrophages, all of which can dampen antitumor immunity.^[^ ^44^ ^]^ This consideration guided our inclusion of a TLR7/8 agonist, 3M‐052, to counteract these immunosuppressive elements.

The integration of a TLR7/8 agonist into our KRAS^G12D^ LNPs aligns with our overarching strategy of enhancing antigen presentation while simultaneously overcoming immune suppression in PDAC.^[^ ^7^, ^45^ ^]^ Transcriptomic analyses in our study revealed a significant upregulation of MHC I‐ and MHC II‐associated components following combination therapy, indicating improved cross‐presentation of tumor antigens to CD8⁺ T cells, as well as enhanced CD4⁺ T cell activation. CD4⁺ T cells provide crucial support for sustaining CTL responses and fostering immune memory.^[^ ^46^, ^47^ ^]^ Moreover, TLR7/8 agonists have been shown to suppress MDSC and Treg generation in the spleen while also repolarizing M2 macrophages toward a pro‐inflammatory, antitumor phenotype.^[^ ^48^, ^49^, ^50^, ^51^ ^]^ Beyond adaptive immunity, TLR7/8 activation stimulates innate immune components, including DCs, macrophages, and NK cells, amplifying the immune response initiated by the KRAS neoantigen vaccine.^[^ ^49^ ^]^ These findings align with prior studies demonstrating that incorporating TLR7/8 agonists, such as R848 and 3M‐052, into nanoparticle formulations can significantly enhance the efficacy of PDAC immunotherapies.^[^ ^7^, ^52^, ^53^ ^]^

Importantly, survival analysis in our orthotopic KPC model demonstrated that the combination therapy significantly extended survival compared to monotherapies or saline controls. This outcome underscores the potential clinical relevance of our approach, as prolonged survival in preclinical models is often indicative of a more effective and durable therapeutic strategy. Mechanistic insights from our gene expression analysis further support the notion that the observed survival benefits are driven by a more robust and coordinated immune response. Specifically, our findings suggest that the ability of this combination therapy to enhance antigen presentation and sustain T cell activation underpins its superior efficacy in controlling tumor progression.

In addition to the therapeutic combination discussed in the current communication, our findings also demonstrate the potential to strengthen the therapeutic outcome of existing immunotherapies, including the use of checkpoint blocking antibodies or small molecule inhibitors addressed in our previous studies.^[^ ^3^, ^6^, ^54^ ^]^ Specifically, we demonstrated that irinotecan‐loaded silicasomes synergize with immune checkpoint inhibitors to enhance therapeutic efficacy in PDAC models.^[^ ^6^ ^]^ We have also developed ICD‐inducing nanocarriers synergizing with next‐generation immune modulators, including remote loading of GSK3 inhibitors and prodrugs interfering in the PD‐1/PD‐L1 axis.^[^ ^3^, ^54^ ^]^ Together, these studies highlight the modularity of our approach and its potential to strengthen the cancer immunity cycle by integrating with existing and evolving immunotherapies. Moving forward, rational combination strategies leveraging our nanoparticle platforms could offer new opportunities to overcome immune resistance and improve clinical outcomes in PDAC and other immunologically cold tumors.

Taken together, our study highlights the potential of a combinatorial nanocarrier‐based strategy as a promising avenue for future PDAC immunotherapies. While monotherapies targeting individual immune pathways have yielded limited success in PDAC, our results emphasize the importance of integrating multiple immune‐stimulating mechanisms to elicit a more comprehensive and durable antitumor response.^[^ ^3^ ^]^ By leveraging both ICD‐driven antigen release and spleen‐targeted immune modulation, our approach provides a strong foundation for the development of next‐generation immunotherapeutic interventions for this highly resistant malignancy.

While our study provides compelling evidence for the efficacy of this combinatorial approach, several important questions remain. First, further investigation is needed to optimize the dosing and timing of the two nanocarrier systems to maximize therapeutic synergy. Additionally, studies assessing the potential for immune memory formation is necessary to determine whether this approach can provide long‐term protection against PDAC recurrence. While this study focuses on therapeutic efficacy in established tumors, future work will include prophylactic vaccination models to assess long‐term memory responses and prevention of recurrence This will be particularly relevant for evaluating our platform as an adjuvant strategy following surgical resection in PDAC. Importantly, transcriptomic evidence of enhanced antigen presentation and T cell activation supports the mechanistic rationale for memory T cell induction, underscoring the translational promise of this approach. While integration of mRNA and chemotherapeutic agents into a single nanocarrier may be conceptually attractive, practical limitations in formulation will interfere in efficient co‐loading due to incompatible physicochemical properties of the cargos. Our dual‐delivery approach maintains the functional integrity of each therapeutic modality, reflecting a clinically relevant strategy that mirrors current combinatorial immunotherapy regimens using separately administered agents to sequentially engage distinct elements of the cancer immunity cycle.^[^ ^55^ ^]^

Another key consideration is the clinical translation of our approach, particularly given the growing momentum behind mRNA‐based cancer immunotherapies.^[^ ^56^, ^57^ ^]^ The success of mRNA vaccine technology, which gained prominence during the COVID‐19 pandemic, has catalyzed significant advancements in oncology. Several efforts now focus on leveraging mRNA to generate robust antitumor immunity, with KRAS‐targeted mRNA vaccines emerging as a promising strategy. For instance, Moderna's mRNA‐5671 (V941) encapsulates mRNA encoding KRAS mutations (G12D, G12V, G13D, and G12C) within LNPs and is currently under clinical evaluation for PDAC, non‐small cell lung cancer, and colorectal cancer. This vaccine is designed to activate CTLs through APC‐mediated presentation, showcasing the potential of mRNA‐based approaches in solid tumors.

In addition to KRAS‐directed vaccines, personalized neoantigen vaccines are demonstrating compelling clinical potential. A recent Phase 1 trial of autogene cevumeran, an individualized neoantigen mRNA–lipoplex vaccine, in combination with atezolizumab (anti‐PD‐L1) and mFOLFIRINOX chemotherapy, reported long‐term immune responses in pancreatic cancer patients.^[^ ^55^ ^]^ Notably, vaccine responders exhibited a prolonged recurrence‐free survival compared to non‐responders, with CD8⁺ T cell clones persisting at substantial frequencies 3 years post‐vaccination. Importantly, these vaccine‐induced T cells exhibited a tissue‐resident memory‐like phenotype with cytotoxic effector function, a characteristic critical for sustained tumor surveillance. Interestingly, autogene cevumeran not only elicits long‐lived neoantigen‐specific CD8⁺ T cells but also utilizes mRNA–lipoplex nanoparticles optimized for TLR7 activation, a mechanism that can further enhance innate immune signaling and antigen presentation. The ability to integrate personalized neoantigen recognition with TLR7‐driven innate immune activation positions this approach as a promising strategy to overcome the immunosuppressive tumor microenvironment in PDAC.

Beyond mRNA vaccines, peptide‐based KRAS immunotherapies have also advanced into clinical trials. For example, the TG01 peptide cocktail, which incorporates multiple KRAS mutant peptides, has been combined with granulocyte‐macrophage colony‐stimulating factor to enhance antigen presentation and immune activation in pancreatic cancer (NCT02261714).^[^ ^58^ ^]^ However, while peptide‐based vaccines offer a potential means of targeting KRAS‐driven malignancies, mRNA‐based platforms provide a more versatile and scalable approach, capable of rapid adaptation to different tumor neoantigens. Given the evidence that autogene cevumeran induces long‐lived T cell responses and TLR7 activation, it further supports the rationale for integrating mRNA‐based vaccination with immune‐stimulatory adjuvants to amplify anti‐PDAC immunity.

While our current study made use of a TLR7/8 agonist to enhance antigen presentation and immune activation, previous studies, including our own, have demonstrated that STING agonists also serve as potent immune adjuvants.^[^ ^5^, ^59^ ^]^ We recently reported the co‐delivery of a STING agonist with mRNA in liver‐targeting nanoparticles to reprogram the tolerogenic environment, highlighting the use of additional immunomodulators to shape the immune responses in cancer.^[^ ^5^ ^]^ We also demonstrated that these particles confer memory T cell responses that can be adoptively transferred. Other studies have explored the use of STING agonists with mRNA in lymph node‐targeting nanoparticles, demonstrating robust tumor‐specific immune responses and long‐term memory in murine models.^[^ ^60^ ^]^ Although our current study did not include a STING agonist, it would be of interest to compare its immunomodulatory effects with 3M‐052, including considering their combined use. Thus, our findings open the door for additional immunomodulatory combinations, including the use of antibody or prodrug checkpoint inhibitors, IDO‐1 and CXCR4 inhibitors, as previously described by us.^[^ ^3^, ^54^, ^61^ ^]^ It is also possible to combine ICD stimuli with CD40 agonists, Flt3L agonists, blockers of the WNT pathway, antibodies that target M2 macrophages (e.g., CSF1R inhibitors) or augment the contribution of NK cells.^[^ ^61^, ^62^, ^63^, ^64^, ^65^ ^]^

## 4 Conclusion

In summary, our study demonstrates that the combination of irinotecan‐loaded silicasomes and spleen‐targeting KRAS mRNA LNPs effectively synergizes to overcome the immunosuppressive barriers of PDAC. By simultaneously inducing ICD, enhancing antigen presentation, and reprogramming the immune environment, this approach successfully bridges innate and adaptive immunity, leading to improved tumor regression and prolonged survival. These findings highlight the potential of nanocarrier‐based strategies to revolutionize PDAC treatment and lay the groundwork for future clinical translation. Ultimately, our work underscores the importance of integrating multiple immune‐activating mechanisms to achieve more effective and durable antitumor immunity in pancreatic cancer.

## 5 Experimental Section

### Materials

DODAP, cholesterol, DOPE, DMG‐PEG_2000_, DSPC, and DSPE‐PEG_2000_ were obtained from Avanti Polar Lipids, USA. PolyA was obtained from Sigma‐Aldrich, Inc. The Luciferase mRNA (mLuc) and mRNA constructs (mRAS^wt^ and mKRAS^G12D^) were ordered from TriLink Biotechnologies. 3M‐052 (Telratolimod) was purchased from Medchem Express, USA. Irinotecan hydrochloride trihydrate was purchased from LC Laboratories, USA. Quant‐iT RiboGreen RNA Kit, DiR dye (DiIC_18_(7), and RNAlater RNA Stabilization Solution were purchased from Thermo Fisher Scientific Inc., USA. Fetal bovine serum (FBS) was purchased from Gemini Bio Products. Matrigel Basement Membrane Matrix was purchased from Corning.

### Synthesis and Characterization of LNPs

LNPs were synthesized by the NanoGenerator Flex‐M system (Precigenome LLC) to blend and mix the lipid‐containing organic phase with an aqueous phase containing mRNA constructs. The aqueous phase was comprised of RNase‐free sodium acetate buffer (0.1 M, pH 4.0) containing the mRNA constructs (PolyA, mLuc, mRAS^wt^, or mKRAS^G12D^). For LNPs/mRNA synthesis, the organic phase was comprised of a lipid mix (DODAP/ DOPE/ cholesterol/ DMG‐PEG_2000_) dissolved in ethanol in a molar ratio of 18.5: 60: 20: 1.5. For 3M‐LNPs/mRNA synthesis, the lipophilic TLR7/8 agonist, 3M‐052, was loaded into the LNP structure by substituting a small fraction of DOPE (0‐ 4.5%) with the lipid agonist. For the 3M‐LNPs/mRNA^G12D^ used in animal experiments, the organic phase was comprised of a lipid mix (DODAP/ DOPE/cholesterol/ DMG‐PEG_2000_/3M‐052) dissolved in ethanol in a molar ratio of 18.5: 55.5: 20: 1.5: 4.5. The flow rate ratio of the aqueous versus organic phase was 3:1. Particles in the effluent phase were dialyzed against PBS (pH 7.4) to remove ethanol before use. The LNPs were characterized for hydrodynamic size, PDI, and zeta potential by a ZETAPALS instrument (Brookhaven Instruments Corporation). The EE% of mRNA constructs were performed through the RiboGreen assay and determined by UV spectroscopy (M5e, Molecular Device, USA). The concentration of 3M‐052 was determined by the UV–vis absorbance at 320 nm.

For the animal imaging studies, DiR‐labeled LNPs (DiR‐LNPs/mLuc and DiR‐3M‐LNPs/mLuc) were used. The synthesis was carried out by the addition of the DiR dye to the organic phase at 0.5% of the total lipid content. The rest of the procedure was as for the LNPs described above.

### Assessment of Biodistribution and Spleen‐Targeting Function of LNPs

The DiR‐labled LNPs (DiR‐LNPs/mLuc and DiR‐3M‐LNPs/mLuc) were prepared for animal imaging studies. The UCLA Animal Research Committee approved all animal experimental protocols. Healthy B6129SF1/J mice received a single IV administration of DiR‐LNP/mLuc or DiR‐3M‐LNPs/mLuc to deliver 0.3 mg kg^−1^ luciferase mRNA (*n* = 3). Mice were intraperitoneally injected with D‐Luciferin 24 h after LNP injection and euthanized within 10 min. Major organs (heart, lungs, kidneys, spleen, and liver) were collected for the subsequent ex vivo IVIS imaging and quantitative analysis of DiR fluorescence.

### Assessment of the Therapeutic Efficacy of the Spleen‐Targeting LNPs in an Orthotopic KPC Pancreatic Cancer Model

A KPC‐derived orthotopic tumor model was established in immunocompetent B6129SF1/J mice, as previously described.^[^ ^6^, ^7^, ^8^ ^]^ An orthotopic KPC model was opted for due to the logistical and experimental limitations inherent to the spontaneous model, including the high cost of maintaining a breeding colony and the asynchronous and variable onset of tumor formation in the transgenic mice. This makes it challenging to accumulate enough tumor‐bearing animals of a defined age to comprehensively execute therapeutic studies. Moreover, the orthotopic model allows for consistent tumor establishment and synchronized treatment initiation, enabling reproducible and statistically powered studies, closely resembling human PDAC. Briefly, A 50 µL suspension of DMEM/Matrigel (6:4 v/v), containing 0.8 × 10^6^ KPC‐luc cells, was used for injection into the pancreas tail of 9‐week‐old female B6129SF1/J mice by a short survival surgery procedure, 6 days prior to treatment. Tumor growth was confirmed by IVIS bioluminescence imaging 1 day prior to commencement of therapy, using tumor‐bearing mice for random assortment into 4 groups (*n* = 6), namely: saline, 3M‐LNP/mRAS^wt^, LNP/mKRAS^G12D^ and 3M‐LNP/mKRAS^G12D^. These animals received IV injection of the different treatment modalities every 3 days on 4 occasions. The different dose equivalents for mRNA and 3M‐052 amounted to 1.25 and 1.75 mg kg^−1^ respectively. Bioluminescence imaging of the luciferase‐expressing tumors was performed on day 5, 11, 14, 18, 25, and 33 by intraperitoneal injection with 50 mg kg^−1^ D‐Luciferin. To assess survival rate, animals were provided with supportive care and monitored regularly until they met euthanasia criteria or expired naturally according to UCLA ARC regulations. The survival results were plotted as Kaplan–Meier curves, followed by data analysis to calculate MST and %ILS compared to saline group. Tumors were collected from animals meeting euthanasia criteria or expired naturally, followed by submerging in 10% formalin for the performance of IHC staining. IHC analysis was performed to assess tumor staining intensity for CD8 and granzyme B. Image processing was performed using Aperio ImageScope software (Leica).

While luciferase expression had been reported to elicit immune responses in murine models, the long‐standing use of the KPC‐Luc system had not revealed significant interference with tumor growth or immune response readouts.^[^ ^66^ ^]^ Moreover, prior studies had concluded that luciferase do not alter tumor biology or immune infiltration patterns.^[^ ^67^, ^68^ ^]^ Luciferase‐expressing KPC cells were also widely used for non‐invasive imaging of orthotopic PDAC tumor model in the investigation of immunotherapy.^[^ ^69^, ^70^ ^]^

### IFN‐γ ELISPOT Assay

Healthy female B6129SF1/J mice (n = 3) received IV injections every 3 days to deliver total of 4 doses of either saline, 3M‐LNP/mRAS^wt^, LNP/mKRAS^G12D^, or 3M‐LNP/mKRAS^G12D^ (mRNA, 1.25 mg kg^−1^; 3M‐052, 1.75 mg kg^−1^). On day 15, mice were sacrificed, and spleens were collected for further analysis. Splenocytes were isolated by passing the spleen tissue through a 70 µm cell strainer, followed by red blood cell lysis using ACK buffer. After washing with PBS, splenocytes were transferred into an IFN‐γ ELISPOT plate and co‐incubated with medium only, wild‐type RAS peptide, and KRAS^G12D^ peptide for 20 h. The plates were washed and treated with a biotinylated detector antibody, followed by incubation with a streptavidin‐alkaline phosphatase mix. Spot formation was visualized using BCIP/NBT substrate solution, following the manufacturer's protocol. After drying, the plate was imaged using an ELISPOT reader (CTL ImmunoSpot), and the number of IFN‐γ spots was quantified.

Assessment of Immune Response in response to combination therapy with 3M‐LNP/mKRAS plus 3M‐Si‐IR in an Orthotopic KPC Pancreatic Cancer Model: A 50 µL suspension of DMEM/Matrigel (6:4 v/v), containing 0.8 × 10^6^ KPC‐luc cells, was injected into the pancreatic tails of 9‐week‐old female B6129SF1/J mice 5 days before the treatment. After establishing the KPC‐luc tumor model and confirming tumor growth by IVIS bioluminescence imaging on day 5, tumor‐bearing mice were randomly divided into 4 groups (n = 4), including saline, 3M‐Si‐IR, 3M‐LNP/mKRAS^G12D^, and 3M‐LNP/mKRAS^G12D^ + 3M‐Si‐IR. Mice received IV injections of 3M‐Si‐IR as priming endogenous immunological stimulus on day 5 and 8, followed by IV injection of the exogenous vaccine booster (3M‐LNP/mRNA^G12D^) on days 12 and 15. Each injection of 3M‐LNP/mKRAS^G12D^ delivered a dose equivalent of 1.25 mg kg^−1^ mRNA and 1.75 mg kg^−1^ 3M‐052, while the dose equivalents for irinotecan and 3M‐052 in 3M‐Si‐IR amounting to 40 and 2 mg kg^−1^, respectively. Bioluminescence imaging of the luciferase‐expressing tumors was performed on days 5, 12, and 18 by intraperitoneal injection with 50 mg kg^−1^ D‐Luciferin. Animals were euthanized and sacrificed on day 18, followed by harvesting of the primary tumors before separation into two parts. One tumor piece was fixed in 10% formalin for the performance of IHC staining for CD8^+^, granzyme B, CRT, IFN‐γ and PD‐1. The other tumor piece was submerged in RNAlater buffer at 2–8 °C overnight, then transferred to –80 °C for bulk RNA sequencing.

### Bulk RNA Sequencing

Frozen tumor samples were processed for RNA extraction and quality control at the Technology Center for Genomics & Bioinformatics (TCGB) at UCLA. RNA concentration and purity were assessed using NanoDrop, while integrity evaluation was performed using TapeStation. Libraries for RNA sequencing were prepared using the KAPA Stranded mRNA‐Seq Kit, and sequencing was performed on the Illumina NovaSeq X Plus with a paired‐end (PE) 2 × 50 run. Data quality checks were conducted using Illumina SAV, while demultiplexing was performed with Illumina Bcl2fastq v2.19.1.403 software. Alignment was carried out using STAR with the Mouse mm39 genome, and gene feature annotation was performed using GRCm39.108.gtf.

Following sequencing and alignment, raw read counts from STAR were normalized using the MRN method to correct for sequencing depth variations. To evaluate the impact of irinotecan‐induced ICD at the tumor site and KRAS mRNA delivery via LNPs to lymph nodes, gene sets related to apoptosis, ER stress, DAMP release, APC activation, cytokine secretion, antigen presentation, T cell activation, and T cell exhaustion were extracted from curated GO terms and the KEGG pathway database.

### Statistical Analysis

Differences among groups were estimated by one‐way ANOVA analysis. Data were expressed as mean ± standard deviation (SD), representing at least three independent experiments. The survival analysis was performed by Log Rank testing (Mantel‐Cox), using GraphPad Prism 10 software. A statistically significant difference was considered at ^*^ *p* < 0.05; ^**^ *p* < 0.01; ^***^ *p* < 0.001; ^#^ *p* < 0.05; ^##^ *p* < 0.01, and ^###^ *p* < 0.001, as indicated in the figure legends.

## Conflict of Interest

Andre E. Nel is a co‐founder and equity holder in Westwood Biosciences Inc. and Nammi Therapeutics. Andre E. Nel also serves on the Board for Westwood Biosciences Inc. The remaining authors declare no conflict of interest.

## Supporting information



Supporting Information

## Data Availability

The data that support the findings of this study are available from the corresponding author upon reasonable request.
