# Supplementary material for: KRAS mRNA Spleen‐Targeting Lipid Nanoparticles Synergize with Irinotecan Silicasomes to Robustly Augment the Cancer Immunity Cycle in Pancreatic Cancer
Source: Adv Sci (Weinh). 2025 Jun 11;12(34):e04886. doi: 10.1002/advs.202504886 (PMC12442683; doi:10.1002/advs.202504886)
Supplement: Supplementary file 1 — Supporting Information [file ADVS-12-e04886-s001.docx]

Supporting Information

The KRAS mRNA Spleen-targeting Lipid Nanoparticles Synergize with Irinotecan Silicasomes to Robustly Augment the Cancer Immunity Cycle in Pancreatic Cancer

Lijia Luo, Xiang Wang, Yu-Pei Liao and Andre E. Nel*

**Figure S1.** Different LNP samples were formulated with varying molar percentages of 3M-052 (0, 0.5, 1.5, 3.0, and 4.5 mol%). The lipid component ratios were detailed, and these formulations were evaluated to determine the optimal 3M-052 molar ratio, as shown in Figure 2. Based on the appropriate 3M-052 and mRNA amounts for animal experiments, we decided to go forward with the particle composition shown in Sample 5 (3M-LNPs containing 4.5% 3M-052).


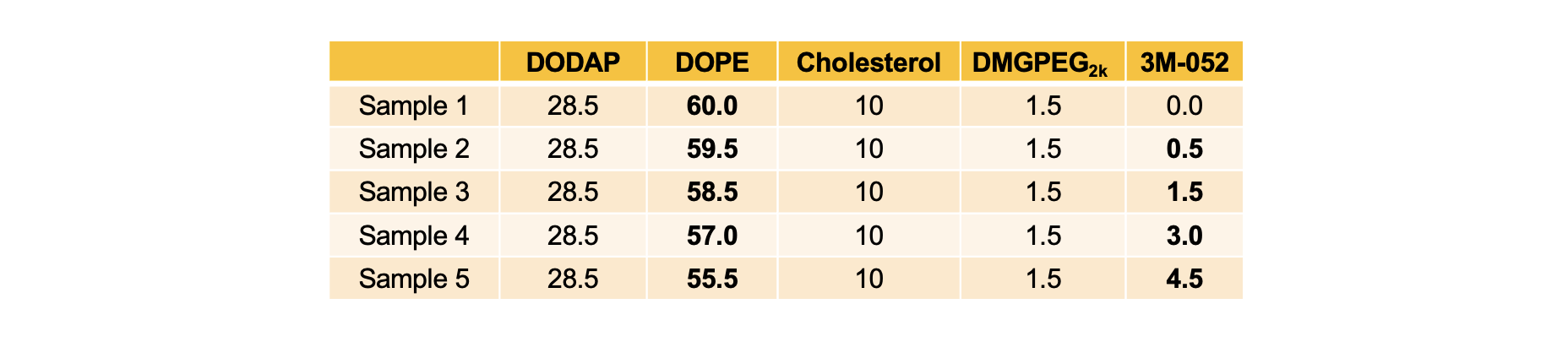


**Figure S2.** Schematic illustration of spleen targeting LNPs, explaining how the DODAP/DOPE composition facilitates spleen targeting through the formation of a protein corona. The protein corona, which consists of proteins adsorbed onto nanoparticles in their biological environment, is strongly influenced by the particle’s lipid composition. DODAP/DOPE-LNPs are advantageous for the spleen targeting by interacting with the C3 complement protein, which serves as a ligand for binding to C3 receptors present on the surface of APC in the spleen. This interaction facilitates particle endocytosis and translation of the KRAS^G12D^ mRNA, allowing for antigen presentation to cytotoxic T cells in the spleen.^[1–4]^ In contrast, LNPs designed for liver targeting typically incorporate ionizable or permanently cationic lipids such as DLin-KC2-DMA, DLin-MC3-DMA, or ALC-0315, which preferentially bind apolipoprotein E (ApoE). This interaction facilitates hepatocyte uptake via LDL receptor-mediated endocytosis. However, the liver’s high enzymatic activity and metabolic turnover contribute to rapid mRNA degradation, limiting its potential for translation. By contrast, spleen-resident dendritic cells provide a more favorable environment for endosomal escape and cytoplasmic release of mRNA, supporting enhanced gene expression. This helps to clarify the results demonstrated in Figure 3.


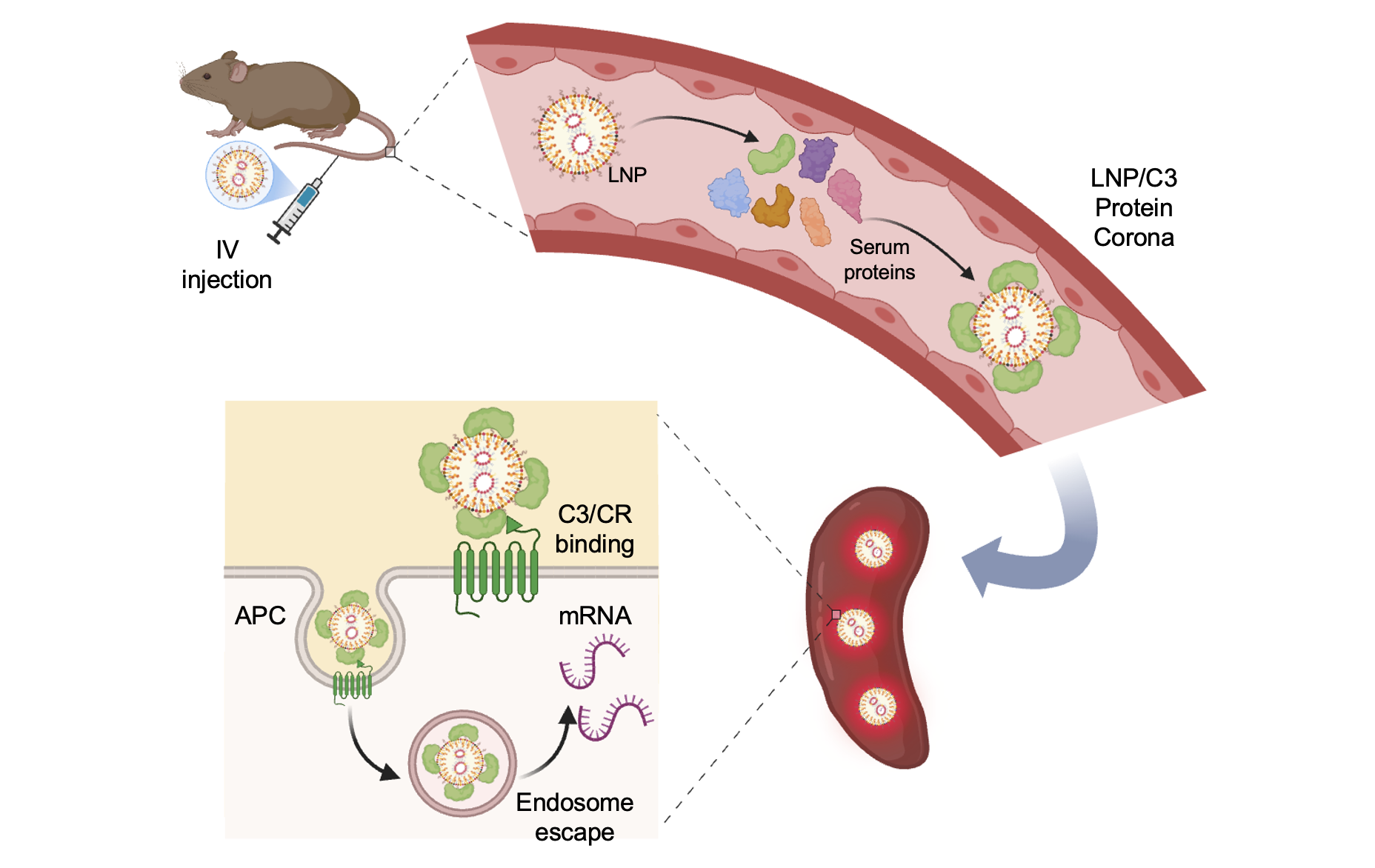


**Figure S3.** Gel analysis of mRAS^wt^ and mKRAS^G12D^ from TriLink Biotechnologies. Figure 4A described the preparation of the mRNA strands for incorporation into a cationic LNP. The mRNA construct features a 5-tandem-repeat nucleotide sequence of RAS_5-21_, flanked by two upstream and downstream amino acids. The ORF consists of a start codon, the epitope nucleotide sequence, and a stop codon, integrated with a 5′Cap, 5′ untranslated region, 3′ untranslated region, and a PolyA tail, forming the complete mRNA sequence. Both strands were synthesized by TriLink Biotechnologies, including performance of gel electrophoresis to demonstrate RNA lengths of ~600 nt.


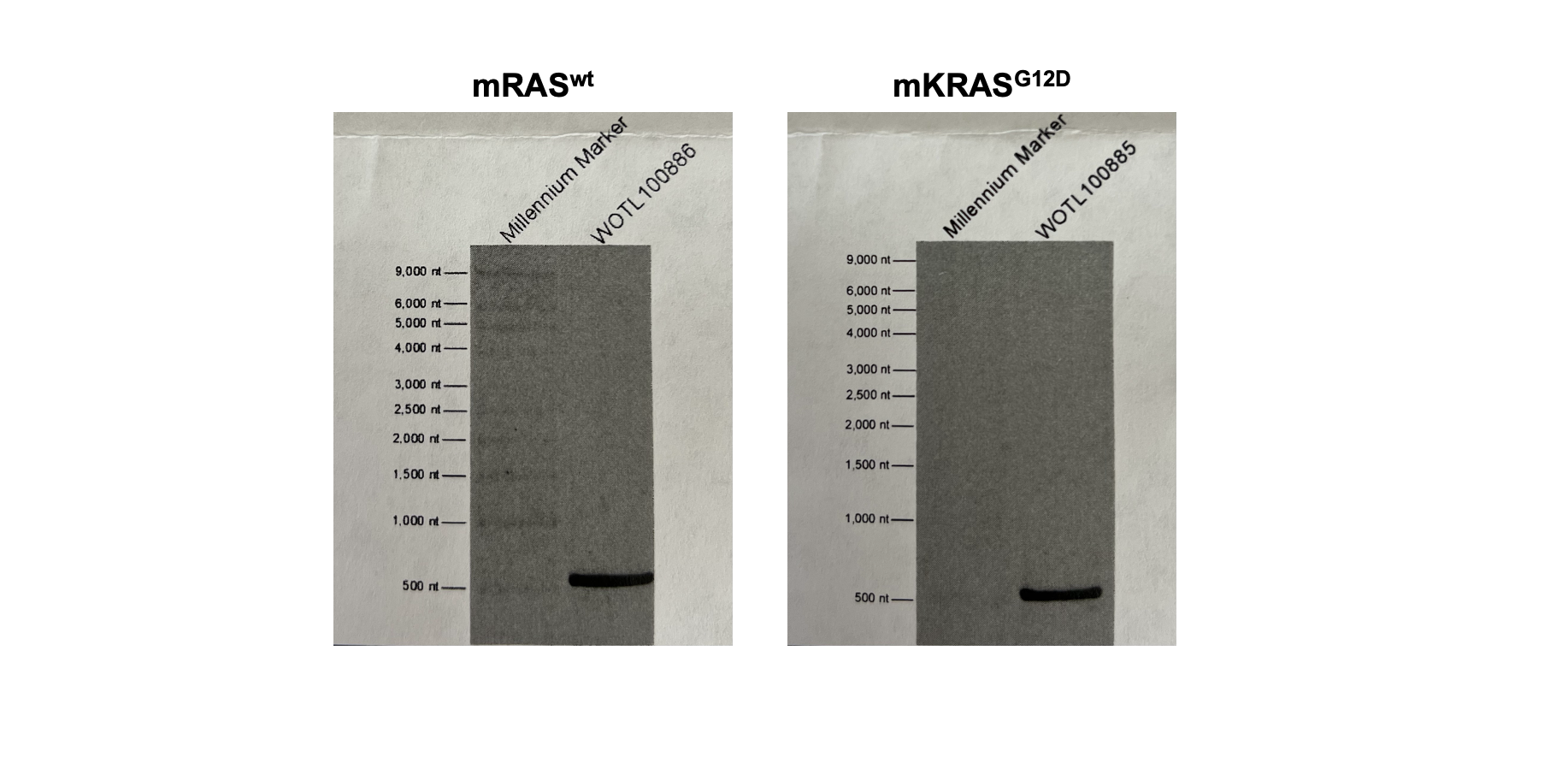


**Figure S4**. Schematic explaining silicasome composition, synthesis and biological impact. A) Schematic explaining the composition of the irinotecan silicasome, 3M-Si-IR, as previously described by us.^[5]^ 3M-052 is a lipid tail TLR 7/8 agonist, which can be incorporated into the lipid bilayer, which encapsulates mesoporous silica nanoparticles (MSNP). Panels A, C and E were reprinted with permission from ref 5. Copyright 2022 American Chemical Society. B) Schematic to explain the mechanism of irinotecan remote loading, utilizing passive loading of the MSNP pores with the trapping agent, TEA_8_SOS, before lipid coating. ^[6–8]^ This allows remote loading of amphipathic irinotecan, crossing the lipid bilayer against the proton gradient established by the trapping agent. Finally, the protonated drug interacted with SOS^8–^ to form a drug precipitate, which is stably entrapped until uptake and delivery into the PDAC tumor site. C) CryoEM visualization of 3M-Si.^[5]^ D) Schematic and electron microscopy demonstration of how silicasomes gain access to the PDAC site by a transcytosis process that depends on endothelial cell endocytic vesicles rather than the frequently cited EPR effect, as previously demonstrated by us.^[9]^ Used with permission of American Society for Clinical Investigation, from ref 9; permission conveyed through Copyright Clearance Center, Inc. E) Demonstration of the improved pharmacokinetics that is achievable by silicasomes versus free drugs.^[5]^ While free irinotecan is rapidly cleared from the blood, the 3M-silicasome-IR significantly increased the circulating plasma irinotecan concentration. The increased circulation time, plus efficient transcytosis, results in multifold increase in the intratumor irinotecan and 3M-052 drug concentrations. F) The generation of ICD markers (CRT) and CD8^+^ T cell infiltration in the primary KPC tumors by the irinotecan silicasome, as described by us.^[7]^ Reprinted with permission from ref 7 under a Creative Commons Attribution License 4.0 (CC BY). Copyright 2021 The Authors.


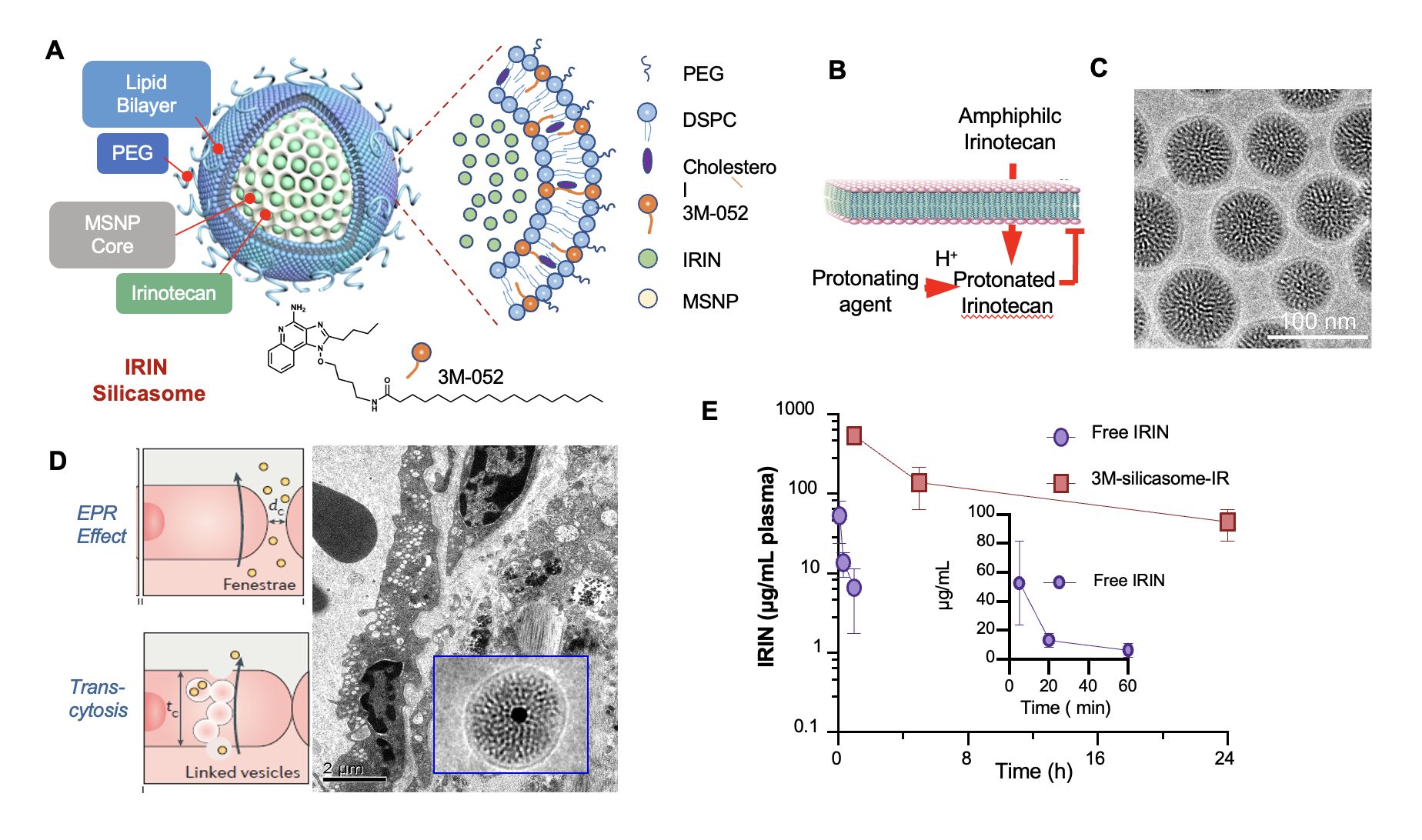


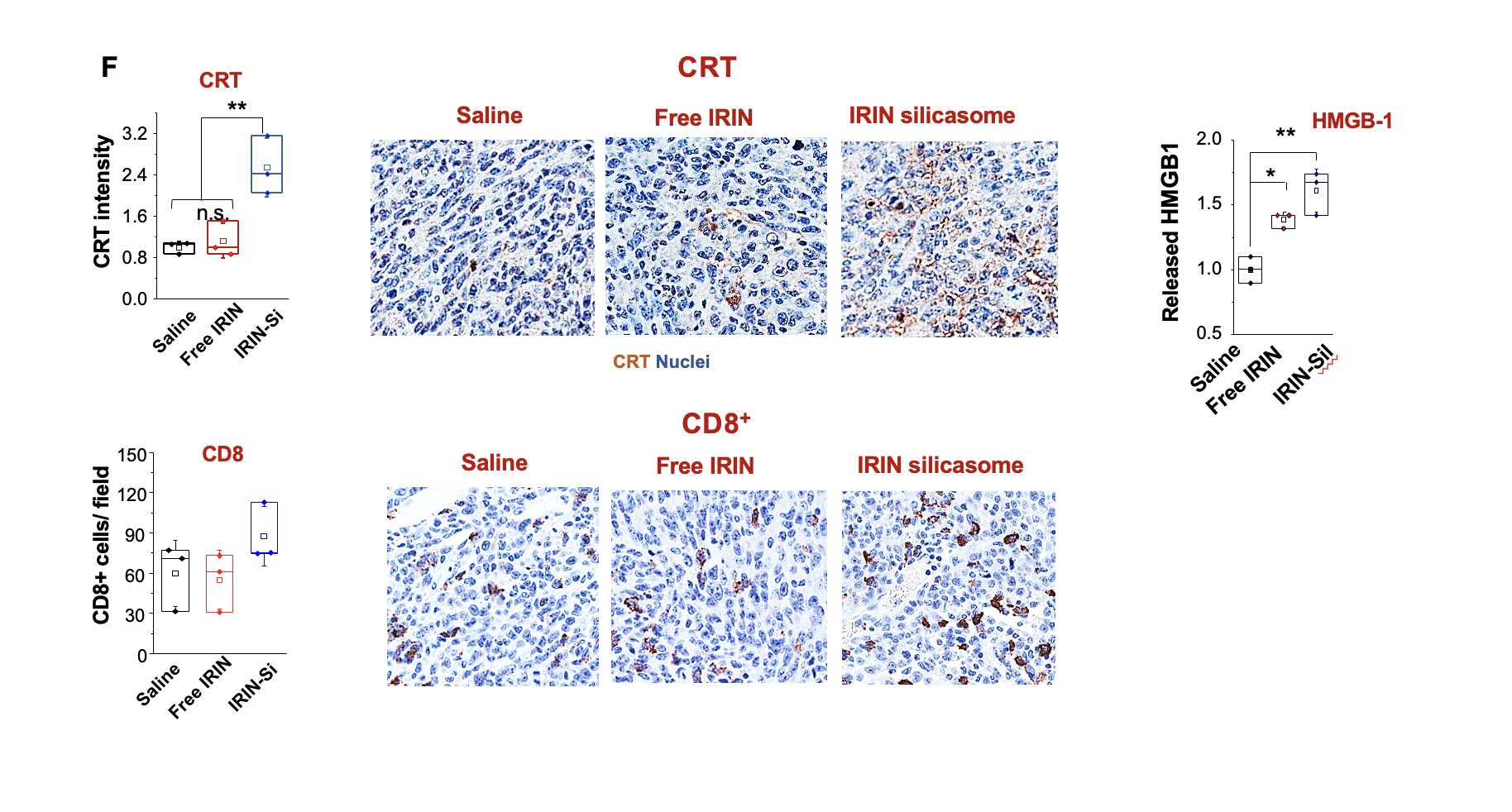


**Figure S5.** Silicasome synthesis and characterization for undertaking the experimentation in Figure 6. The nanoparticles with synthesized as previously described by us.^[5]^ Briefly, MSNP was first soaked in TEA_8_SOS at 65 °C before being added to preheated ethanol containing dissolved 3M-052 and lipids (DSPC/Chol/DSPE-PEG2000/3M-052, 55.5:38.5:2.7:3.3 molar ratio). The mixture was sonicated to form a LB coating. For irinotecan loading, irinotecan was dissolved in HEPES-buffered dextrose and incubated with purified TEA_8_SOS-loaded 3M-silicasome at 65 °C. The process was quenched in ice water, followed by purification of the 3M-silicasome-IR (3M-Si-IR). Subsequent physicochemical characterization of 3M-Si-IR demonstrated the size, polydispersity index (PDI), zeta potential, irinotecan loading capacity (EE%), and the CryoEM image as depicted. The scale bar is 100 nm.


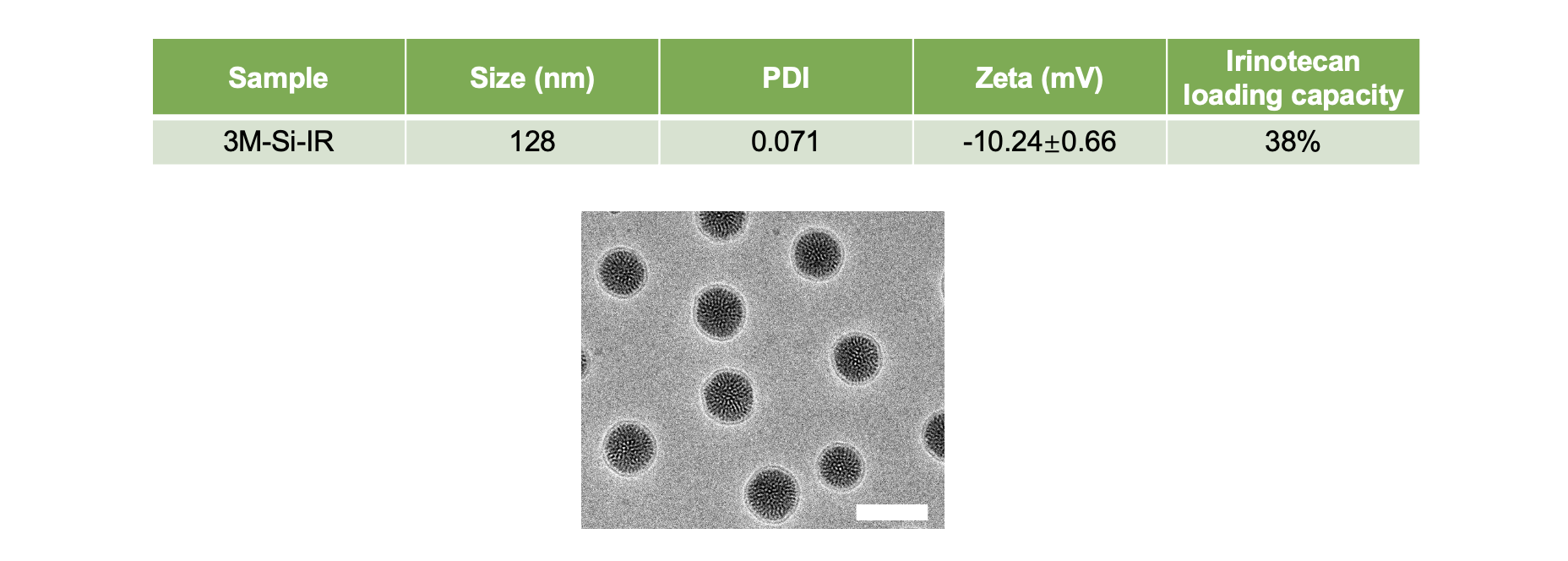


**Figure S6.** To explore the potential impact of the sequence of administration, we implemented a partially altered dosing schedule to perform a survival experiment in which the 3M-LNP/mKRAS^G12D^ was initiated prior to Si-IR, followed by coupled co-administration. The survival experiment used a dual delivery in which the order of administration was partially reversed, compared to the experiments described in Figure 6 and 7. Orthotopic KPC tumors were established in animals (n =6), followed by twice-weekly IV injections of either saline or 3M-LNP/mKRAS^G12D^ (mKRAS^G12D^, 1.25 mg/kg; 3M-052, 1.75 mg/kg) for two weeks starting on day 5. This was followed by twice-weekly IV injections of either saline or 3M-Si-IR (IRIN, 40 mg/kg) for an additional two weeks beginning on day 9 animals were monitored daily until reaching moribund status or spontaneous death. Kaplan–Meier survival analysis revealed that Si-IR (p<0.001), and 3M-LNP/mKRAS^G12D^ (p<0.001) significantly extended survival, with the greatest benefit seen in the 3M-LNP/mKRAS^G12D^ + Si-IR group (p<0.001, yielding median survival time: of 42.5 days, % ILS: 117.9%). Please notice that while this experiment does not represent a full reversal of the sequence, the observed immunologic and combinatorial effects still show preserving of therapeutic synergy despite the partial variation in dosing order. Further studies are warranted to systematically evaluate the influence of administration sequence on treatment outcomes.


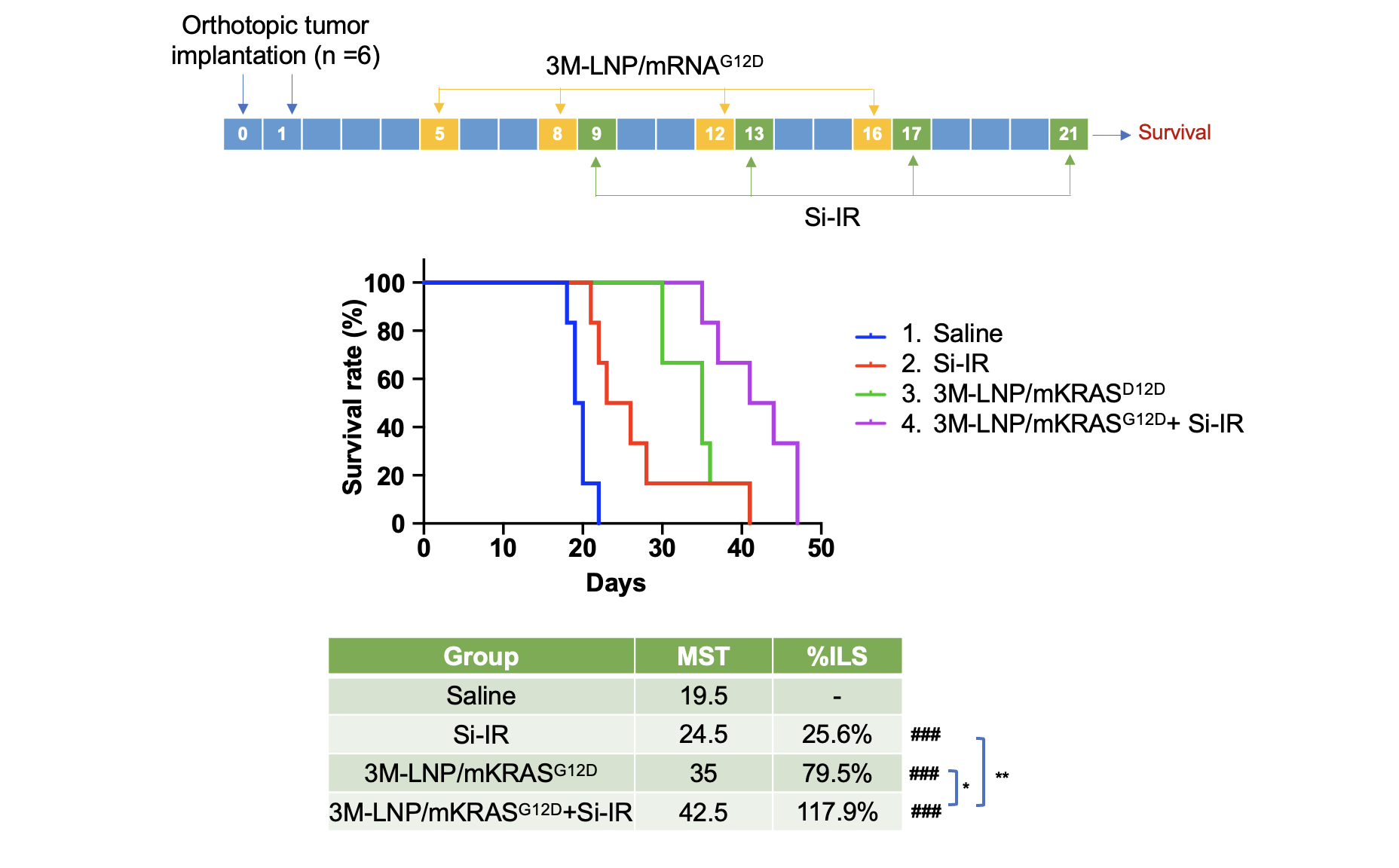


**Figure S7.** Heatmap analysis of an additional 9 key genes associated T cell activation. The accompanying protein functions are also included. The transcriptomic analysis was undertaken as described in Figure 8.


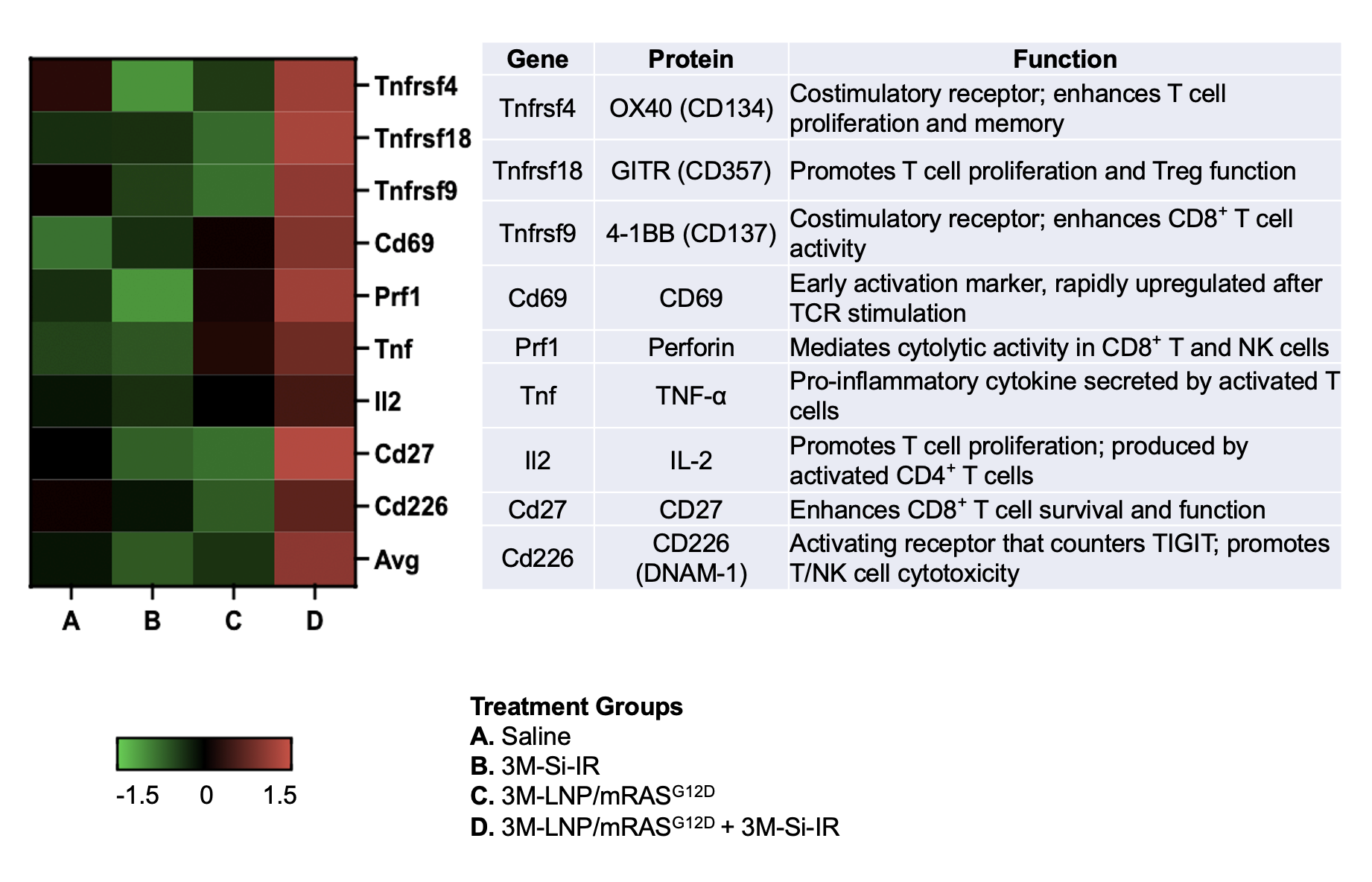


**Figure S8.** Heatmap analysis of 8 key genes associated with immune checkpoint receptors. The relevant proteins and their function are also included. The transcriptomic analysis was undertaken as described in Figure 8.


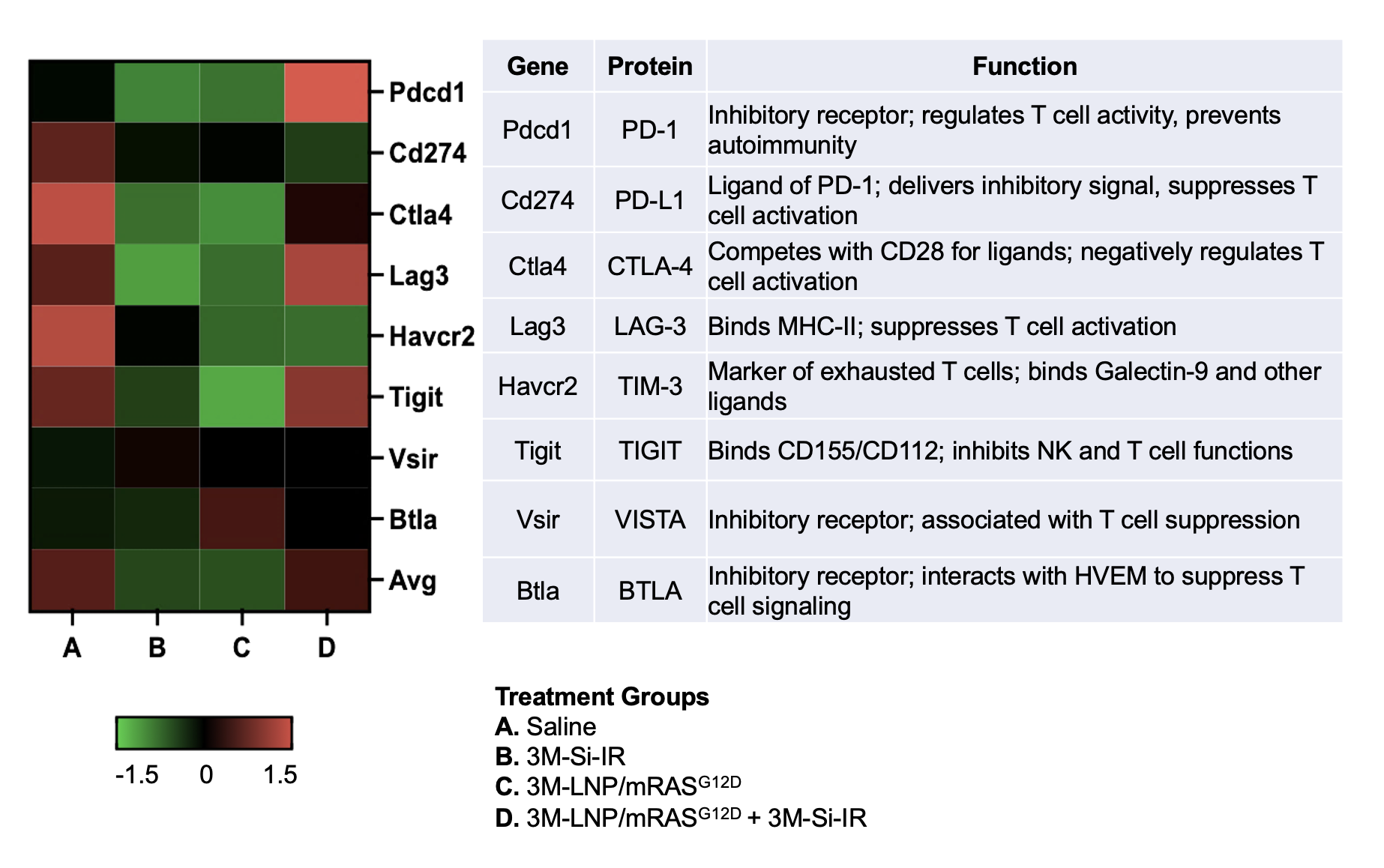


**Figure S9.** Heatmap analysis of 8 key genes associated with T cell transcription factors. The relevant proteins and their function are also included. The transcriptomic analysis was undertaken as described in Figure 8.


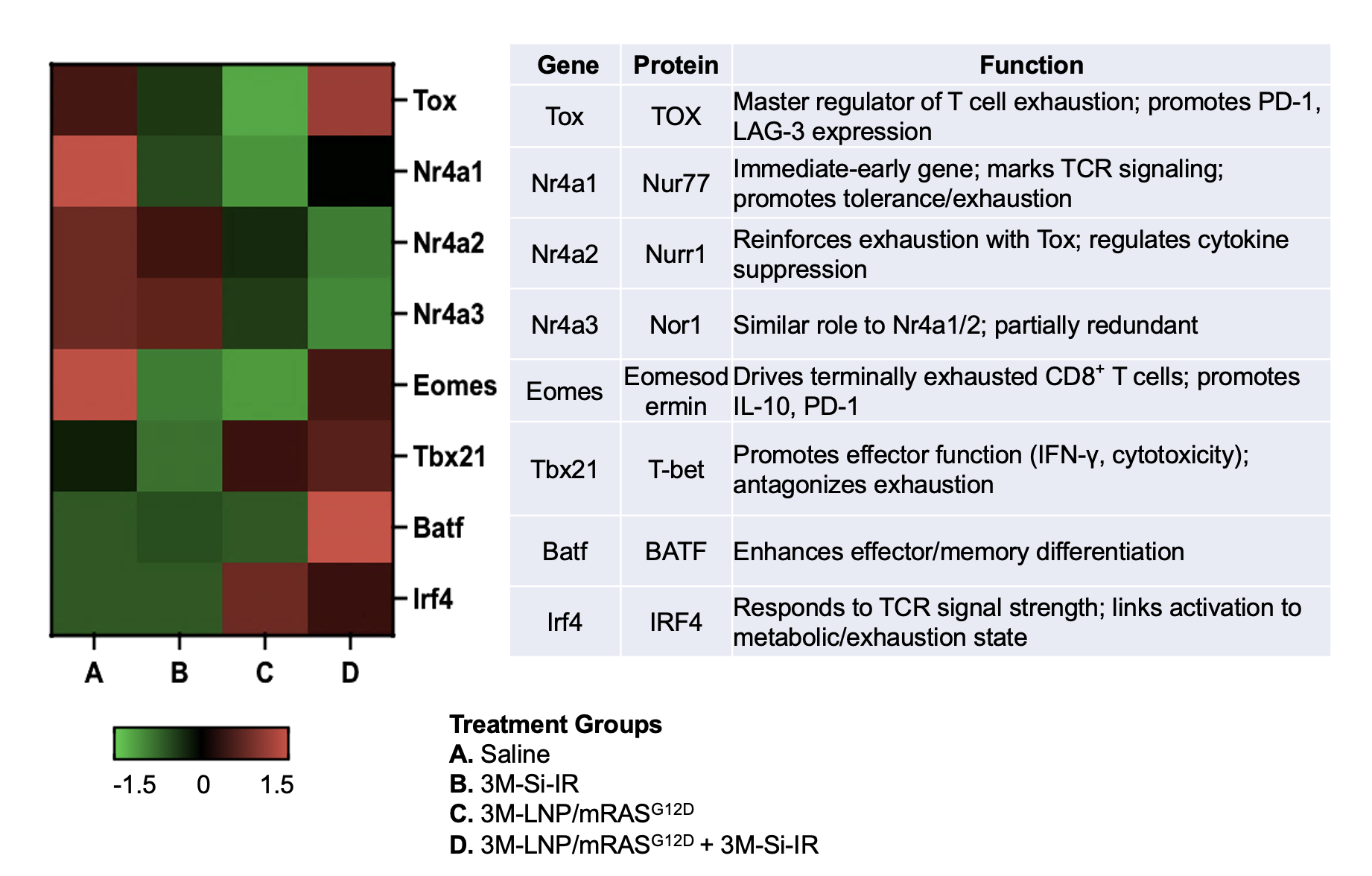


**References**

[1] Liu, Y., Zhang, R., Qiu, N., Wang, S., Chen, J., Xu, X., Xiang, J., et al., Spleen‐Targeted mRNA Nanoparticles for Modulating B Cell Hyperactivation in Rheumatoid Arthritis Therapy, 2024, Adv. Funct. Mater., 35, 2417101, <https://doi.org/10.1002/adfm.202417101>

[2] Mold, C., Effect of membrane phospholipids on activation of the alternative complement pathway, 1989, J. Immunol., 143, 1663, <https://doi.org/10.4049/jimmunol.143.5.1663>

[3] Kimura, S., Khalil, I.A., Elewa, Y.H.A., & Harashima, H., Novel lipid combination for delivery of plasmid DNA to immune cells in the spleen, 2021, J. Control. Release, 330, 753, <https://doi.org/10.1016/j.jconrel.2021.01.005>

[4] Shimosakai, R., Khalil, I.A., Kimura, S., & Harashima, H., mRNA-Loaded Lipid Nanoparticles Targeting Immune Cells in the Spleen for Use as Cancer Vaccines, 2022, Pharmaceuticals, 15, 1017, <https://doi.org/10.3390/ph15081017>

[5] Luo, L., Wang, X., Liao, Y.-P., Chang, C.H., & Nel, A.E., Nanocarrier Co-formulation for Delivery of a TLR7 Agonist plus an Immunogenic Cell Death Stimulus Triggers Effective Pancreatic Cancer Chemo-immunotherapy, 2022, ACS Nano, 16, 13168, <https://doi.org/10.1021/acsnano.2c06300>

[6] Liu, X., Situ, A., Kang, Y., Villabroza, K.R., Liao, Y., Chang, C.H., Donahue, T., et al., Irinotecan Delivery by Lipid-Coated Mesoporous Silica Nanoparticles Shows Improved Efficacy and Safety over Liposomes for Pancreatic Cancer, 2016, ACS Nano, 10, 2702, <https://doi.org/10.1021/acsnano.5b07781>

[7] Liu, X., Jiang, J., Liao, Y.-P., Tang, I., Zheng, E., Qiu, W., Lin, M., et al., Combination Chemo-Immunotherapy for Pancreatic Cancer Using the Immunogenic Effects of an Irinotecan Silicasome Nanocarrier Plus Anti-PD-1, 2021, Adv. Sci., 8, 2002147, <https://doi.org/10.1002/advs.202002147>

[8] Liu, X., Jiang, J., Chan, R., Ji, Y., Lu, J., Liao, Y.-P., Okene, M., et al., Improved Efficacy and Reduced Toxicity Using a Custom-Designed Irinotecan-Delivering Silicasome for Orthotopic Colon Cancer, 2019, ACS Nano, 13, 38, <https://doi.org/10.1021/acsnano.8b06164>

[9] Liu, X., Lin, P., Perrett, I., Lin, J., Liao, Y.-P., Chang, C.H., Jiang, J., et al., Tumor-penetrating peptide enhances transcytosis of silicasome-based chemotherapy for pancreatic cancer, 2017, J. Clin. Invest., 127, 2007, <https://doi.org/10.1172/JCI92284>
